# Nonclinical Safety Evaluation of Renogrit: In Vitro Nonmutagenicity (OECD 471) and 28‐Day Repeated Oral Dose Toxicity (OECD 407) in Sprague Dawley Rats

**DOI:** 10.1155/jt/5905101

**Published:** 2026-04-24

**Authors:** Acharya Balkrishna, Aakanksha Tiwari, Himanshu Jangid, Kamaraj Mani, Savita Lochab, Sandeep Sinha, Anurag Varshney

**Affiliations:** ^1^ Drug Discovery and Development Division, Patanjali Research Foundation, Haridwar, India, patanjaliresearchfoundation.com; ^2^ Department of Allied and Applied Sciences, University of Patanjali, Haridwar, India, universityofpatanjali.com; ^3^ Patanjali UK Trust, Glasgow, UK

**Keywords:** Ames test, ayurveda, mutagenicity, NOAEL, OECD 407, Renogrit, toxicity

## Abstract

Renogrit is a prescription medicine developed by employing the traditional knowledge of Ayurveda for the management of kidney disorders. To support its extensive clinical investigations, Renogrit requires robust nonclinical safety assessments. Accordingly, in this study, in vitro mutagenicity assay and in vivo subacute toxicity were conducted as per the Organization for Economic Co‐operation and Development (OECD) guidelines. Mutagenic potential of Renogrit was tested using *Salmonella typhimurium* and *Escherichia coli uvrA* tester strains in the presence and absence of metabolic activation. DMSO stock solution of Renogrit was assessed at 0.05, 0.15, 0.5, 1.5, and 5.0 mg/plate concentrations, in triplicates, along with the vehicle (DMSO) control and respective positive controls. The revertant colonies were counted after 64‐ to 72‐h incubation period at 37°C. Renogrit was administered to Sprague Dawley (SD) rats by oral route for 28 consecutive days, at the dose levels of 100, 300, and 1000 mg/kg/day. Animals were monitored for all major toxicological parameters, such as morbidity and mortality, clinical signs, body weight, feed consumption, ophthalmological examinations, and functional observational battery (FOB) assessments during the live phase of the study. At study termination, all animals were subjected to hematological analysis, clinical chemistry analysis, examination of organs for gross pathology, and histopathological investigations. The revertant colonies counted in the Renogrit‐incubated plates were not significantly increased when compared to vehicle‐treated plates, thereby signifying its nonmutagenic potential. Additionally, the subacute toxicity study revealed no toxicologically significant changes attributable to Renogrit administration up to a dose of 1000 mg/kg/day. In conclusion, Renogrit was found to be a nonmutagenic at the evaluated concentrations, and its No Observed Adverse Effect Level (NOAEL) was determined to be 1000 mg/kg/day. The study outcomes provide future nonclinical safety assessments of Renogrit and its detailed clinical evaluation.

## 1. Introduction

Drug‐induced nephrotoxicity remains a significant clinical challenge, often limiting the therapeutic utility of several essential pharmacological agents. Traditional herbal medicines have long been recognized for their nephroprotective potential, offering a complementary approach to managing renal injury. Renogrit, an Ayurvedic prescription medicine, comprises the hydromethanolic extracts obtained from different parts of botanical drugs (Table 1) and has been developed by the traditional wisdom of Ayurveda for the management and treatment of an assortment of kidney‐related disorders.

**TABLE 1:** Composition of Renogrit tablet.

S. no.	Vernacular name	Botanical name	Family	Extract	Quantity per tablet (mg)
1	Apamarg	*Achyranthes aspera* L.	Amaranthaceae	Root extract	71.5
2	Pashanbhed	*Saxifraga ligulata* Murray	Saxifragaceae	Root extract	71.5
3	Palash	*Butea frondosa* Roxb. ex Willd	Fabaceae	Flower extract	71.5
4	Varun	*Crataeva nurvala* Buch.‐Ham	Capparaceae	Bark extract	71.5
5	Punarnavamool	*Boerhavia diffusa* L.	Nyctaginaceae	Root extract	71.5
6	Kasni	*Cichorium intybus* L.	Asteraceae	Whole plant extract	53.0
7	Kasni	*Cichorium intybus* L.	Asteraceae	Seed extract	18.0
8	Gokharu	*Tribulus terrestris* L.	Zygophyllaceae	Fruit extract	71.5

*Note:* Excipients: Gum acacia, talcum, microcrystalline cellulose, and croscarmellose sodium have been used for formulating the Renogrit tablet.

Among the several phytoconstituents present in Renogrit, Apamarg (*Achyranthes aspera*) is a versatile botanical drug used in Ayurveda for its detoxifying and diuretic properties. Jayakumar et al. reported that it helps in the removal of toxins from the body, particularly through the urinary system, thereby promoting renal health [1]. Apamarg is also known for its ability to support the healthy functioning of the kidneys [2] by managing the formation of kidney stones and helping in their dissolution. Additionally, it helps in managing infections and inflammation in the urinary tract [3, 4].

Koul, et al. documented that Pashanbhed (*Saxifraga ligulata*), another component of Renogrit, is a well‐known Ayurvedic botanical drug for its effectiveness in managing kidney stones [5] and promoting renal health. It acts as a natural lithotriptic, helping to break down stones in the kidneys and urinary tract, facilitating their easy passage [6]. Pashanbhed also possesses diuretic properties [7], which increase urine output and help flush out toxins from the kidneys. Its anti‐inflammatory and antibacterial properties help in managing inflammation and infections in the renal system [8, 9].

Palash (*Butea frondosa*), also present in Renogrit, is an important botanical drug, valued for its renoprotective and diuretic properties. It supports the elimination of waste products from the body by enhancing kidney function. Palash is also effective in managing urinary tract infections and the formation of kidney stones. Krolikiewicz‐Renimel et al. published that it has anti‐inflammatory and detoxifying effects which help in maintaining the overall health of the kidneys and urinary system [10].

Another component of Renogrit, namely, Varun (*Crataeva nurvala*), is a potent botanical drug known for its ability to support kidney health and manage urinary disorders [11]. It helps in breaking down and eliminating kidney stones and managing their recurrence. Cho et al. reported that Varun also manages the flow of urine and the symptoms of urinary tract infections [12]. It is commonly used in Ayurveda to manage conditions, such as cystitis, bladder disorders, and renal calculi, due to its anti‐inflammatory and diuretic properties.

Punarnavamool (*Boerhavia diffusa*) is a renowned botanical drug with powerful diuretic and rejuvenating properties [13]. Karwasra et al. declared that it is widely used to support kidney function and promote detoxification through the urinary system. Punarnavamool helps in managing swelling and inflammation in the kidneys, making it effective in managing chronic kidney conditions [14]. It also helps in flushing out excess fluids and toxins from the body, which helps in maintaining a healthy renal system.

Kasni (*Cichorium intybus*), another phytoconstituent of Renogrit commonly known as chicory, is a botanical drug known for its hepatoprotective properties [15], and An et al. published its renal‐supportive properties [16]. It helps in detoxifying the liver and kidneys, thereby promoting their healthy function. Kasni is beneficial in managing urinary tract infections and the formation of kidney stones [17]. Its diuretic properties help in increasing urine output, helping to flush out toxins and maintain the overall health of the kidneys.

Gokharu (*Tribulus terrestris*) is a popular botanical drug, widely used in Ayurveda, and is known for its diuretic and kidney‐supportive properties [18]. It is effective in managing kidney stones and their formation. Shetty et al. reported that Gokharu also supports the overall health of the urinary tract by managing inflammation and infections [19]. It enhances urine flow, helping in the elimination of toxins from the body [20], and is commonly used in the management of various renal disorders [21].

Ultra‐high‐performance liquid chromatography (UHPLC) analysis of Renogrit, from our previously reported research, ascertained that Renogrit is enriched with several bioactive phytometabolites, known to possess beneficial effects in kidney disorders. According to the published article by Balkrishna et al., these metabolites include gallic acid, bergenin, methyl gallate, quercetin, and boeravinone [22]. The amount of the bioactive phytometabolites present in each tablet of Renogrit as ascertained from the previous study is elaborated in Table 2.

**TABLE 2:** Phytometabolites identified and quantified in Renogrit tablet by UHPLC analysis.

S. no.	Compound name	Quantity present Renogrit (μg/mg)	Total quantity (μg/tablet)
1	Gallic acid	2.47	1333.8
2	Bergenin	8.88	4795.2
3	Methyl gallate	1.86	1004.4
4	Quercetin	0.17	91.8
5	Boeravinone B	0.05	27

*Note:* Information excerpted and modified from Reference [23].

Abbreviation: UHPLC, ultra‐high‐performance liquid chromatography.

Balkrishna et al. reported that the pharmacological effects of Renogrit have been previously tested in our laboratory against vancomycin‐induced kidney injury in both in vitro and in vivo experiments.

In vitro experiments, such as cell viability, function of P‐glycoprotein, KIM‐1, NAG levels, and mRNA expression using q‐RT‐PCR for MMP‐7 and NGAL, were conducted in spheroids of HK‐2 cells (human proximal tubular cell) induced by vancomycin. Incubation of Renogrit with HK‐2 cells positively modulated all the molecular biomarkers to prove the promising activity at the cellular level. In vivo model of vancomycin‐induced nephrotoxicity in Sprague Dawley (SD) rats was conducted. Renal function parameters, such as blood urea nitrogen (BUN), creatinine (CREAT), and their clearances, were estimated as a primary efficacy parameter. The terminal parameters, such as eGFR, relative kidney weight, GSH/GSSG ratio, and histology of kidney, were also performed. To strengthen the efficacy of Renogrit, calculated molecular biomarkers viz. KIM‐1, NAG, and osteopontin were quantified. Treatment of Renogrit protected and normalized the renal abnormalities of kidney functions induced by vancomycin to show encouraging efficacy on renal health. The outcomes of these experiments established that Renogrit possesses potential for alleviating vancomycin‐associated nephrotoxicity [22].

Furthermore, our laboratory has also demonstrated the protective activity of Renogrit in cisplatin‐induced human renal proximal tubular (HK‐2) cells and *Caenorhabditis elegans*. Balkrishna et al. demonstrated that Renogrit treatment decreased renal tubular cell injury by mitigating cisplatin‐induced oxidative stress, mitochondrial dysfunction, apoptosis, necroptosis, mitophagy, and inflammation by targeting multiple pathways of cell injury, without affecting the anticancer potential of cisplatin. The renoprotective potential of Renogrit was evident from its ability to regulate renal injury markers, redox imbalance, and mitochondrial dysfunction. Further, Renogrit modulated apoptosis, necroptosis, mitophagy, and inflammation [23].

Taking into account the well‐characterized pharmacological effects of Renogrit, in both in vitro and in vivo studies, it is considerably imperative to establish its nonclinical safety as well, with a broad objective of providing its detailed preclinical and subsequently clinical investigations. Consequently, in this study, we have evaluated the mutagenic potential of Renogrit in vitro as well as its tolerability after 28‐day repeated administration to SD rats, in accordance with the Organization for Economic Co‐operation and Development (OECD) Guidelines. The mutagenic potential of Renogrit was assessed using the bacterial reverse mutation (Ames) test according to the OECD Test Guideline 471 [24] by the plate incorporation method, in the absence of metabolic activation, while the pre‐incubation method was followed in the presence of metabolic activation. The reverse mutation was assessed in four strains of histidine‐dependent *Salmonella typhimurium* in addition to tryptophan‐dependent *Escherichia coli WP2 uvrA* tester strains. Test bacterial culture was incubated with different concentrations of Renogrit (0.05, 0.15, 0.5, 1.5, and 5.0 mg/plate) or standard mutagens, in the absence or presence of S9 mix comprising of 10% S9 fraction. DMSO was used as the vehicle control for these experiments. The revertant (visible) colonies were counted after the incubation period. Mutagenicity was then determined based on an increase in revertant colonies.

Further, the subacute toxicity of Renogrit was conducted after its repeated administration by the intragastric route to male and female SD rats, for an overall duration of 28 days. This study was performed in conformance with OECD Test Guideline 407 [25]. The primary objective of this study was to identify any treatment‐related major toxicological effects, determine the target organs, and establish the No Observed Adverse Effect Level (NOAEL) for Renogrit in rats. Our study design also incorporated a satellite group of animals that received the high dose of the test article, similarly, for 28 consecutive days, after which the test article administration was discontinued. Subsequently, the animals allocated to this particular arm of the study were then continuously monitored for an additional 14 days to assess the reversibility, persistence, and delayed occurrence of toxicologically relevant effects post‐treatment cessation. Parameters evaluated during the in‐life phase of the study and at study termination included clinical observations, body weight monitoring, feed consumption pattern, hematological and biochemical parameters, relative organ weights, and gross and histopathological evaluations of all the harvested organs. The outcomes of both of the experiments should be valued in addition to the systemic safety of profiling of the Ayurvedic medicine, Renogrit, for further development and clinical utility.

## 2. Materials and Methods

### 2.1. Test Item, Chemicals, and Reagents

Renogrit (internal batch number: PRF/CHI/0323/0407), a free‐flowing, light‐brown powder, intended for the preparation of granules and ultimately for compression into a tablet formulation, was sourced from Divya Pharmacy, Haridwar, India. *S. typhimurium* tester strains, namely TA 98 (Cat. No.: # BAA 2720), TA 100 (Cat. No.: # BAA 2721), TA 1537 (Cat. No.: # 29630), and TA 1535 (Cat. No.: # 29629), and *E. coli* tester strain *WP2 uvrA* (Cat. No.: # 49979) were procured from the American Type Culture Collection (ATCC), USA. Phenobarbital (PB)‐ and β‐naphthoflavone (BNF)‐induced S9 fractions from male SD rat liver homogenate (Cat. No.: # 11‐05L.2) were procured from MOLTOX®, Molecular Toxicology, Inc., NC, USA. Other purchased reagents for Ames test include bacteriological agar powder (Cat. No.: # GRM026, HiMedia, India), L‐histidine (Cat. No.: # GRM050, HiMedia, India), nutrient broth (Cat. No.: # M1362, HiMedia, India), D‐biotin (Cat. No.: # TC096, HiMedia, India), glucose‐6‐phosphate (Cat. No.: # 32994, SRL, India), NADPH (Cat. No.: # 11407, Spectrochem, India), magnesium chloride hexahydrate (Cat. No.: # MB040, HiMedia, India), KCl (Cat. No.: # P9311, Sigma‐Aldrich, USA), 4‐nitroquinoline N‐oxide (Cat. No.: # N8141, Sigma‐Aldrich, USA), sodium azide (Cat. No.: # S8032, Sigma‐Aldrich, USA), 9‐aminoacridine (Cat. No.: # A2905, Tokyo Chemical Industries, India), dimethyl sulfoxide (DMSO, Batch No.: # M545932503, Loba Chemie), and 2‐aminoanthracene (Cat. No.: #A38800, Sigma‐Aldrich, USA). Methyl cellulose (MC) (Cat. No.: # 04635) was purchased from Loba Chemie, India. A 0.5% aqueous solution of MC was used as a vehicle for preparing the desired concentrations of Renogrit suspensions. Tropicamide eyedrops (1%) were bought from Akums Drug and Pharmaceuticals Ltd., India. Thiopentone sodium injection (THIOSOL™ sodium) was obtained from Neon Laboratories Ltd., India. Thymol crystals (Cat. No.: # 32196) were procured from Molychem, Mumbai, India. Sodium citrate (Cat. No.: # 83407) was purchased from Sisco Research Laboratories Pvt. Ltd., India, whereas ethylenediaminetetraacetic acid (EDTA) dipotassium salt (Cat. No.: # E0270), Giemsa stain (Cat. No.: # 48900), and methylene blue (Cat. No.: # M9140) were bought from Sigma‐Aldrich, USA. Reagents used for preparing 10% neutral‐buffered formalin (NBF) include sodium phosphate dibasic (Cat. No.: # S0400, RANKEM), sodium phosphate monobasic (Cat. No.: # S0240., RANKEM), and formaldehyde (Cat. No.: # 1.04003, Merck). Reagents for preparation of modified Davidson’s fixative include glacial acetic acid (Cat. No.: # A0060 RANKEM), ethanol (Cat. No.: # 1170, Changshu Hongsheng Fine Chemical Co. Ltd.), and formaldehyde (Cat. No.: # 1.04003, Merck). Reagents used for preparing hematoxylin & eosin staining solution include eosin yellow (Cat. No.: # GRM1060) from HiMedia, India; mercury (II) oxide red (Cat. No.: # 1.93680); potassium aluminum sulfate dodecahydrate (Cat. No.: # 1.93699), procured from EMPLURA, Millipore; and hematoxylin (Cat. No.: # H9627), which was bought from Sigma‐Aldrich, USA.

### 2.2. Ethics Statement and Animal Welfare

The testing facility, Patanjali Research Foundation, Haridwar, is certified by the Committee for Control and Supervision of Experiments on Animals (CCSEA; Registration Number: 1964/PO/Rc/S/17/CPCSEA), Department of Animal Husbandry and Dairying, Ministry of Fisheries, Animal Husbandry and Dairying, Government of India, for conducting experiments on small laboratory animals. The experimental protocol (Proposal No. PRIAS/LAF/IAEC‐149) was meticulously reviewed and received approval from the Institutional Animal Ethics Committee (IAEC) of Patanjali Research Foundation. All animal husbandry practices and humane procedures employed throughout the study were strictly in compliance with the CCSEA guideline as outlined [26]. The study is being reported as per ARRIVE (Animal Research: Reporting In Vivo Experiments) guidelines [27].

### 2.3. Source of Animals and Husbandry Conditions

Sixty‐four SD rats (6–7 weeks old; 32 males and 32 females) were procured from Hylasco Biotechnology Pvt. Ltd., Telangana, India (CCSEA; Registration Number: 1808/PO/RcBt/S/15/CPCSEA), a Charles River Laboratories (Wilmington, Massachusetts, USA)‐licensed domestic animal supplier. Upon receipt, animals were quarantined in a dedicated room for 7 days. Rats were housed in polypropylene cages (Vishnu Traders, Haridwar, India; cage dimensions: 421 × 290 × 190 mm) with sterilized corn cob bedding (Sparcobb™, Sparconn Life Sciences, India). Animals were provided *ad libitum* access to a gamma‐irradiated (25 kGy) standard pelleted laboratory diet (Purina 5L79 Rodent Lab diet, USA) and reverse osmosis water, treated with ultraviolet light and supplied in autoclaved polypropylene bottles. Environmental conditions in the animal facility were maintained at a temperature of 19.0°C–23.0°C and relative humidity between 50% and 70%. The air exchange rate was controlled at 10–15 changes per hour, with a 12‐h light/dark cycle. To minimize stress and associated variability, no more than three animals were housed per cage throughout the study. Cage rotation was performed weekly to ensure uniform environmental exposure across all groups. Animals were individually identified by tail marking, and cage cards displaying detailed group information were used to maintain accurate identification.

### 2.4. In Vitro Bacterial Mutagenicity Assay

*Ames Test: S. typhimurium* tester strains, namely TA 98, TA 100, TA 1537, and TA 1535, and *E. coli* tester strain *WP2 uvrA* were grown overnight at 37°C by inoculating the freshly thawed strains in nutrient broth media. Mutagenicity was assayed by the plate incorporation method in the absence of metabolic activation, while the pre‐incubation method was followed in the presence of metabolic activation as per the OECD Test Guideline 471 [24]. The reverse mutation was assessed in four strains of histidine‐dependent *S. typhimurium* in addition to tryptophan‐dependent *E. coli WP2 uvrA*. The S9 metabolic activation mixture (S9 mix) was prepared according to the OECD Test Guideline 471 [24] and previously published protocols [28, 29]. For the conduct of the experiment, 100 μL of test bacterial culture (1 × 10^9^ cells/mL) was incubated at 37°C with different concentrations of Renogrit or standard mutagen, in the absence or presence of S9 mix comprising of 10% S9 fraction, 5 mM of glucose‐6‐phosphate, 4 mM of NADPH, 8 mM of MgCl_2_, 33 mM of KCl, and 0.1 M phosphate buffer (at pH 7.4) for 30 min, without shaking. Subsequently, 2 mL of soft agar (0.6% bacto‐agar, 0.5% NaCl, 50 μM biotin, and 50 μM histidine for *S. typhimurium* strains or 50 μM tryptophan for *E. coli* strain) was added and poured immediately onto a plate of minimal agar (1.5% bacto‐agar, Vogel‐Bonner E medium, containing 0.4% glucose [GLU]). The standard mutagens, namely 4‐nitroquinoline, at concentration of 0.15 μg/plate were used in TA 98 incubated plates; sodium azide at concentration of 0.5 μg/plate was used in TA 100 and TA 1535 incubated plates; 9‐aminoacridine at concentration of 50 μg/plate was used in TA 1537 incubated plate, and 4‐nitroquinoline at concentration of 0.5 μg/plate was used in *E. coli uvrA* incubated plate without S9 metabolic activation, while 2‐aminoanthracene at a concentration of 20 μg/plate was used with S9 metabolic activation, for all the strains tested. DMSO was used as the vehicle control for these experiments. The mutagenic effect of Renogrit (DMSO stock solution) was assessed at 0.05, 0.15, 0.5, 1.5, and 5.0 mg/plate concentrations, in triplicates, along with the vehicle (DMSO) control and respective positive controls, as per the OECD Test Guideline 471 [24].

The revertant (visible) colonies were counted after 64‐ to 72‐h incubation period at 37°C. A positive result for mutagenicity was determined based on an increase in revertant colonies showing mutagenicity ratio, MR ≥ 2.0, in at least one of the tester strains compared to [24]. The MR was calculated by dividing the number of revertant colonies in Renogrit or the standard mutagen‐treated plates by the number of revertant colonies in the vehicle control (DMSO) plates.

### 2.5. In Vivo 28‐Day Subacute Toxicity

The nonclinical safety evaluation of Renogrit was conducted according to the OECD Test Guideline Number 407 [25]. All female animals were nulliparous and nonpregnant at the time of acquisition. Following a quarantine period, a total of 32 males and 32 females were transferred to an experimental room earmarked for conducting the study. Following a comprehensive clinical examination, all animals were individually weighed using an analytical balance (Model: QUINTIX 1102‐10IN, Sartorius, Germany). Thirty male and 30 female rats were selected for inclusion in the study. Animals were then randomized on the basis of their body weights to six study groups, following the randomization within blocks strategy, using a Microsoft Excel sheet. Each group comprised five males and five females. The sample size was decided based on the recommendations stipulated in the OECD Test Guideline 407 [25]. The experimental design and group allocation of the in vivo subacute toxicity study are presented in Table 3. Two control groups were allocated, namely Group G1: main study control; Group G1R: recovery control. Group G2 animals were assigned to the low‐dose level of 100 mg/kg/day, and Group G3 animals were assigned to the mid‐dose level of 300 mg/kg/day. Two groups, namely G4 and G4R, were assigned to the high‐dose levels of 1000 mg/kg/day (G4, high dose; and G4R, high‐dose recovery, respectively). The body weight variation among animals of either sex did not exceed ±20% of the respective group mean. After randomization, all selected animals underwent an ophthalmoscopic examination using an ophthalmoscope (Model: M2N13000, HEINE Optotechnik GmbH & Co., Germany) after subjecting them to mydriasis with 1% tropicamide solution. Thereafter, animals were subjected to a 5‐day acclimatization period under standard laboratory conditions. During this period, individual rats were identified using tail markings to facilitate differentiation.

**TABLE 3:** Experimental design of in vivo subacute toxicity study.

Group no.	Group	Treatment	Number of animals	Dose (mg/kg/day)	Concentration of formulation prepared (mg/mL)
	**28-day treatment group**				

G1	Control	Vehicle	5M + 5F	0	0
G2	Low dose	Renogrit	5M + 5F	100	10
G3	Mid‐dose		5M + 5F	300	30
G4	High dose		5M + 5F	1000	100

	**14-day recovery group**				

G1R	Control	Vehicle	5M + 5F	0	0
G4R	High dose	Renogrit	5M + 5F	1000	100

*Note:* M denotes male animals, and F signifies female animals; G refers to the group.

The required quantity of Renogrit was accurately weighed using an analytical balance (Sartorius QUINTIX 224‐10IN, Germany) and transferred to a clean mortar. The powder was triturated thoroughly with a pestle to achieve a fine consistency. A calculated volume of 0.5% methylcellulose (MC) solution, used as the vehicle, was then gradually added while continuously triturating to ensure complete wetting of the powder. Subsequently, the remaining volume of MC was added dropwise with continued trituration to obtain a stable and homogeneous suspension. The final dose formulations were prepared at concentrations of 10, 30, and 100 mg/mL, corresponding to target rat doses of 100, 300, and 1000 mg/kg/day, respectively, administered at a dose volume of 10 mL/kg. All formulations were freshly prepared on each day of Renogrit administration to maintain consistency and stability. Animals in the treatment groups received the Renogrit formulations by oral gavage at dose levels of 100 mg/kg/day (Group G2, low dose), 300 mg/kg/day (Group G3, mid‐dose), and 1000 mg/kg/day (Group G4, high dose; and Group G4R, high‐dose recovery). Control groups (G1: main study control; G1R: recovery control) received the vehicle, 0.5% MC. As 0.5% MC was used as a vehicle for Renogrit, the highest dose volume to be given among the experimental group animals was considered, and the same volume of 0.5% MC was administered to all control group animals (G1 and G1R) as recommended by the guideline [25].

### 2.6. Body Weight and Feed Consumption

Body weights were recorded for all animals using an analytical balance (Model: QUINTIX 1102‐10IN, Sartorius, Germany) before the first administration of the test substance and subsequently at weekly intervals throughout the dosing and recovery phases. In the main study arm, animals were weighed on Days 1, 8, 15, 22, and 28. In the recovery arm, additional measurements were taken on Days 35 and 42. Fasting body weights were also recorded on the day of scheduled necropsy for all animals in both the main and recovery groups. Body weight changes were calculated by subtracting the initial (Day 1) body weight from the respective weekly values, enabling assessment of net weight gain or loss over the study period. Feed consumption was also monitored weekly throughout the dosing and recovery phases. Weekly feed consumption per animal was calculated based on the difference as per the given formula, and the data were tabulated accordingly.

(1) Feed consumption per week g=Feed offered g−Feed leftover gNumber of animals .

### 2.7. General and Detailed Clinical Sign Observations

All animals were subjected to daily cage‐side clinical observations for the detection of any abnormal signs. Additionally, animals were monitored twice daily for morbidity and mortality throughout the study duration. Following 28 days of oral administration of Renogrit, animals in the vehicle control recovery group (G1R) and the high‐dose recovery group (G4R; 1000 mg/kg/day) were observed for an additional 14‐day recovery period. Clinical observations focused on the onset, severity, duration, and potential reversibility of any signs of toxicity. All assessments were conducted in accordance with the Guide for the Care and Use of Laboratory Animals (8th Edition, National Research Council, 2011; National Academies Press).

Detailed clinical observations were conducted once weekly for all animals in both the treatment and recovery groups, continuing through to the scheduled termination of the study. Animals were carefully examined for any alterations in skin, fur, eyes, and mucous membranes, as well as for the presence of abnormal secretions or excretions. Basic autonomic functions were also assessed, including lacrimation, piloerection, pupil size, and respiratory patterns [25].

Additionally, animals were observed for changes in gait and posture, reactivity to handling, and the presence of clonic or tonic convulsions. Behavioral assessments included the detection of stereotypic or unusual behaviors, such as excessive grooming, head shaking, circling, or other abnormal activities [25].

### 2.8. Functional Observational Battery (FOB)

FOB assessment [30] was performed during the 4^th^ week in all animals and during the 6^th^ week in the recovery group animals. Motor activity was evaluated using an actophotometer system (Instrument Name: IR Actimeter, Model: LE8825, Panlab, Spain). Before testing, all animals were acclimatized to the testing environment and the activity chamber (dimensions: 45 × 45 × 35 cm) for 60 min per day over four consecutive days. During the third week of the study, animals were allowed to habituate to the testing room for 1 hour for three consecutive days while remaining in their home cages before being individually transferred to the activity chamber. During the fourth week of the study, each animal was then placed in the activity chamber for a duration of 5 min. Locomotor activity was quantified as total movement counts using an IR Actimeter. The apparatus consists of a two‐dimensional square frame (*X* and *Y* axes), each equipped with a 16 × 16 array of infrared beams for animal detection. The infrared photocell array enables precise adjustment to accurately detect rat movements, which were directly captured using a control unit. The number of rearings was, however, recorded manually during the 5‐min observation period. Rat standing upright on its hind limbs, with or without forepaw contact against a wall, was considered a rearing activity. Activity chambers were thoroughly cleaned with a damp cloth and tissue paper between animals to prevent olfactory cues or cross‐contamination. Behavioral, neurological, and autonomic responses were assessed in accordance with standard FOB procedures:

Behavioral Observations: Animals were individually assessed for the behavioral activity. Each animal was taken from the home cage and allowed to freely move in the confined open space. Ease of removal from the cage was initially noted. During the free movement, the animal was observed for normal behavioral responses, such as posture, startle response, alertness, general mobility, fur condition, approach response, touch response, and finally the ease of handling the animal. Responses of the rat based on the above screening were documented for analysis.

Neurological Observations: Animals were individually evaluated for behavioral activity. Each animal was gently removed from its home cage and placed in a confined open‐field arena, where it was allowed to move freely. Neurological behavioral assessments, such as the presence of convulsions or tremors, muscle tone during movement, gait abnormalities, stereotypic or unusual movements, tail pinch response, eye blink response, landing foot splay, and air‐righting reflex, were noted.

Landing Foot Splay Test: A clean sheet of white paper on a flat surface was kept. Hind paws of the animal were coated evenly with ink by impressing them onto a stamp pad. Subsequently, the animal was held approximately 30 cm above the paper. Then, the rat was gently dropped onto the paper; so, the hind paws make contact and left prints. The animal was allowed to step off the paper after landing. The distance (in millimeters) between the inner surfaces of the fourth digits of the hind limbs from the paw print was measured. The procedure was repeated for a total of three consecutive trials per animal. The mean value of the three measurements for each animal was then calculated.

Autonomic Observations: The autonomic behavioral response was observed for individual animals in a confined open field. The responses, such as palpebral closure, were closely watched; lacrimation was observed by the presence of wetness in the eyelid area; salivation was observed by the presence of wetness in the mouth area; color of the left and right eyes was closely observed for any abnormality; mini torchlight was applied in front of the eye; the pupil reflex in response to light was noted; gently, animal was handled to observe the skin color; the presence of piloerection was after close observation of the fur; and the mouth and nose area of the animal were observed for quite some time to evaluate the easiness of respiration and finally allowed the rat to defecate for evaluating the consistency of the faces. All FOB parameters were systematically recorded and tabulated for analysis.

### 2.9. Ophthalmological Examination

With an objective of identifying any treatment‐related changes, ophthalmological examinations were conducted during the fourth week of the study in animals allocated to all groups of both the main study and recovery arms. In addition, the rats assigned to the recovery arm were subjected to ophthalmic examination in the sixth week of study, to assess any delayed onset of ocular effects. Mydriasis was induced using a 1% tropicamide ophthalmic solution, following which both eyes were examined by a trained veterinarian using an ophthalmoscope (Model: M2N13000, HEINE Optotechnik GmbH & Co., Germany) to identify any treatment‐related ocular abnormalities [25].

### 2.10. Hematology and Coagulation

On Day 28 (main study groups) and Day 42 (recovery groups), animals were fasted overnight. Blood samples were collected the following morning (Days 29 and 43, respectively) from the retro‐orbital sinus under transient isoflurane anesthesia. Samples were collected for evaluation of hematological, coagulation, and clinical chemistry parameters [31] to assess potential effects of Renogrit administration. For hematological analysis, blood samples were collected into tubes containing 10% EDTA dipotassium salt, as an anticoagulant. After gentle mixing, samples were analyzed using an automated veterinary hematology analyzer (Model: BC‐5000 Vet, Mindray, Shenzhen Mindray Animal Medical Technology Co., Ltd., China). The following parameters were measured [25]: red blood cell (RBC) count, hematocrit (HCT), mean corpuscular volume (MCV), hemoglobin concentration (HGB), mean corpuscular hemoglobin (MCH), mean corpuscular hemoglobin concentration (MCHC), platelet count (PLT), and white blood cell (WBC) count. Differential leukocyte counts and reticulocyte enumeration were performed manually on blood smears stained with Giemsa and new methylene blue stains, respectively. The percentage of reticulocytes was calculated using the following mathematical formula:

(2) Reticulocyte%=Total reticulocytes1000 RBCs×100.

Whole blood for coagulation studies was collected into vials containing 3.2% sodium citrate. Plasma was separated by centrifugation at 1000 × *g* for 10 min at 4°C using a refrigerated centrifuge (Model: Sorvall Legend Micro 21R Microcentrifuge, Thermo Scientific, USA). Coagulation parameters were analyzed using a semi‐automated coagulation analyzer (Model: ECL‐105; single‐channel coagulation analyzer, Erba Lachema, Czech Republic). The following parameters were assessed: prothrombin time (PT) and activated partial thromboplastin time (aPTT).

### 2.11. Clinical Chemistry

Whole blood samples from overnight‐fasted animals were collected into plain tubes without anticoagulant and allowed to clot undisturbed at room temperature for 1 hour. Subsequently, samples were centrifuged at 1000 × *g* for 10 min at 4°C using a refrigerated centrifuge (Sorvall Legend Micro 21R Microcentrifuge, Thermo Fisher Scientific, USA). Serum was carefully separated and transferred into clean vials for analysis. Clinical chemistry parameters were measured in serum using the fully automated random access clinical chemistry analyzer (Model EM200, Transasia Bio‐Medicals Ltd., India). The evaluated analytes included aspartate transaminase (AST), alanine transaminase (ALT), alkaline phosphatase (ALP), total bilirubin (BILT), blood urea level (BUL), calculated BUN, CREAT, GLU, total cholesterol (CHOL), Total Protein (PRO), albumin (ALB), and protein‐to‐albumin ratio (PAR) [25]. Serum electrolytes, including sodium (Na^+^) and potassium (K^+^), were analyzed using an electrolyte analyzer (Model: EC 90, Erba Lachema, Czech Republic).

### 2.12. Gross Pathological and Histopathological Investigation

Animals were humanely sacrificed by intraperitoneal administration of thiopentone sodium (150 mg/kg). All the rats were subjected to a comprehensive macroscopic pathological examination, which incorporated an exhaustive scrutiny of the external surface of the body, all orifices, and the cranial, thoracic, and abdominal cavities with their content. Following gross examination, selected organs including the liver, kidneys, adrenals, testes or ovaries, thymus, spleen, brain, and heart were carefully dissected, trimmed free of adhering tissues, and weighed. Paired organs were weighed collectively. The organs designated for weighing were subsequently fixed in 10% NBF, except for the eyes and testes, which were fixed in modified Davidson’s fixative.

Relative organ weights were calculated for each animal and expressed as a percentage of both terminal fasting body weight and brain weight using the following formulas:

(3) Relative organ weight w.r.t. body weight %=Absolute organ weight gTerminal body weight g×100,

(4) Relative organ weight w.r.t. brain weight %=Absolute organ weight gAbsolute brain weight g×100.

Additional tissues were harvested from all animals and fixed in 10% NBF for subsequent histopathological evaluation. These included the aorta, femur (including bone marrow), cerebrum, cerebellum, midbrain, epididymis, esophagus, large intestine, lungs, mesenteric lymph nodes, pancreas, sciatic nerve, pituitary gland, seminal vesicle, prostate, skeletal muscle, skin, small intestine, stomach, spinal cord, thyroid and parathyroid glands, trachea, urinary bladder, vagina, uterus, and mammary glands [25].

Tissues from animals in the vehicle control group (G1) and the high‐dose Renogrit group (G4; 1000 mg/kg/day) of the main study arm underwent standard in‐house histological procedures. The samples from preserved organs and tissues were processed for dehydration, clearing, and penetration using a tissue processor (Model: TP 1020, Leica Biosystems, India) and were subsequently embedded in paraffin by using embedding station (Model: HistoCore Arcadia H‐C, Leica Biosystems, India). Thereafter, 3–5 μm thick sections were obtained using a microtome (Model: RM 2245, Leica Biosystems, India) and floated in a tissue flotation bath (Model: Tsgp‐10, Thermo Fisher Scientific, USA) at 55°C–57°C. Further, the sections were transferred to glass slides and stained with hematoxylin–eosin stain. The stained sections were then examined microscopically by using a compound light microscope (Model: AxioScope‐A1, Carl Zeiss, China), and representative images were captured by using AxioVision software. The stained sections were then examined microscopically for any treatment‐related histopathological changes.

### 2.13. Statistical Analysis

Data are presented as mean ± standard deviation (SD) for all experimental groups. Statistical analyses were conducted at a significance level of 5% (*p* < 0.05) using GraphPad Prism software (Version 8.0.2). To assess the statistical significance in the mutagenicity experiment, Student’s *t*‐test was performed with *p* < 0.05 considered significant. Body weight, body weight gain, and feed consumption data were evaluated using two‐way analysis of variance (ANOVA) followed by Tukey’s multiple comparison test. Motor activity, landing foot splay, hematology, clinical chemistry, and organ weight data were analyzed using one‐way ANOVA followed by Dunnett’s multiple comparison test for the main study groups, and Student’s *t*‐test was applied for comparisons within the recovery groups.

## 3. Results

### 3.1. Mutagenicity Assessment

#### 3.1.1. Revertant Colonies and MR

The mutagenic potential of Renogrit was tested in the bacterial reverse mutation (Ames) test using histidine‐dependent *S. typhimurium* tester strains, TA 98, TA 100, TA 1537, and TA 1535, and tryptophan‐dependent *E. coli uvrA* in the presence and absence of metabolic activation with rat liver S9 fraction. Renogrit was tested at concentrations of 0.05, 0.15, 0.5, 1.5, and 5.0 mg/plate. The experimental outcomes demonstrated that the revertant colony count of Renogrit‐treated plates was similar to the DMSO control plate. However, MR of all the standard mutagen‐treated plates ranged between 3.0 and 16.6 reflecting the method’s validity.

Hence, it can be inferred that Renogrit did not demonstrate any mutagenic effect in any of the *S. typhimurium* and *E. coli uvrA* strains up to the concentration of 5.0 mg/plate, both in the absence or presence of metabolic activation. A summary of revertant colony counts obtained in Renogrit and mutagenicity assay in the absence and presence of metabolic activation is presented in Tables 4 and 5, respectively.

**TABLE 4:** Bacterial mutagenicity analysis of Renogrit in the absence of metabolic activation (‐S9).

Renogrit dose (mg/plate)	Bacterial strains									
	TA 1535		TA 1537		TA 98		TA 100		*E. coli uvrA*	
	Mean ± SD^#^	MR	Mean ± SD^#^	MR	Mean ± SD^#^	MR	Mean ± SD^#^	MR	Mean ± SD^#^	MR
DMSO	15.0 ± 3.0	1.0	39.0 ± 9.6	1.0	20.7 ± 0.6	1.0	93.0 ± 2.6	1.0	36.7 ± 3.2	1.0
0.05	12.3 ± 1.5	0.8	40.7 ± 14.0	1.0	16.7 ± 4.2	0.8	122.3 ± 19.4	1.2	39.7 ± 1.5	1.1
0.15	13.0 ± 7.2	0.9	24.7 ± 10.2	0.6	18.7 ± 3.2	0.9	94.3 ± 5.7	1.0	35.0 ± 3.5	1.0
0.5	19.7 ± 5.5	1.3	41.7 ± 12.9	1.1	21.3 ± 0.6	1.0	107.3 ± 4.5	1.2	37.0 ± 4.6	1.0
1.5	15.0 ± 2.6	1.0	35.0 ± 18.7	0.9	20.3 ± 5.9	1.0	103.7 ± 11.2	1.1	28.7 ± 10.7	0.8
5.0	11.7 ± 2.9	0.8	13.3 ± 6.8	0.3	21.3 ± 1.5	1.0	94.0 ± 5.0	1.0	33.7 ± 2.5	0.9
Standard mutagen^Ψ^	184.7 ± 13.5	12.3^∗^	236.3 ± 26.7	6.1^∗^	176.0 ± 6.0	8.5^∗^	338.0 ± 27.5	3.6^∗^	292.3 ± 3.2	8.0^∗^

*Note:* Each value represents mean of revertant colonies ± SD, *n = *3. Bacterial mutagenicity analysis of Renogrit showing the average number of revertants per plate in *S. typhimurium* TA 1535, TA 1537, TA 98, and TA 100 and *E. coli uvrA* strains in the absence of metabolic activation (‐S9). MR, mutagenicity ratio = number of revertant colonies in Renogrit or mutagen/number of revertant colonies in vehicle (DMSO).

Abbreviation: SD, standard deviation.

^Ψ^Standard mutagen: 4‐nitroquinoline (0.15 μg/plate) in TA 98; sodium azide (0.5 μg/plate) in TA 100 and TA 1535, 9‐aminoacridine (50 μg/plate) in TA 1537, and 4‐nitroquinoline (0.5 μg/plate) in *E. coli uvrA.*

^∗^denotes significant (*p* < 0.05) compared to vehicle (DMSO).

^#^ denotes mean of revertant colonies with standard deviation (SD).

**TABLE 5:** Bacterial mutagenicity analysis of Renogrit in the absence of metabolic activation (+S9).

Renogrit dose (mg/plate)	Bacterial strains									
	TA 1535		TA 1537		TA 98		TA 100		*E. coli uvrA*	
	Mean ± SD^#^	MR	Mean ± SD^#^	MR	Mean ± SD^#^	MR	Mean ± SD^#^	MR	Mean ± SD^#^	MR
DMSO	17.7 ± 3.8	1.0	13.3 ± 3.8	1.0	22.7 ± 7.8	1.0	113.0 ± 4.4	1.0	47.3 ± 6.0	1.0
0.05	22.3 ± 6.7	1.3	17.3 ± 2.5	1.3	24.7 ± 8.7	1.1	111.7 ± 4.2	1.0	31.0 ± 19.2	0.7
0.15	17.0 ± 1.7	1.0	21.3 ± 11.0	1.6	23.3 ± 3.1	1.0	113.0 ± 4.4	1.0	37.3 ± 3.5	0.8
0.5	18.3 ± 0.6	1.0	18.0 ± 7.9	1.4	19.0 ± 3.0	0.8	110.7 ± 8.6	1.0	48.0 ± 28.8	1.0
1.5	18.3 ± 2.3	1.0	22.7 ± 6.8	1.7	22.7 ± 2.9	1.0	125.0 ± 24.4	1.1	30.3 ± 16.9	0.6
5.0	16.7 ± 1.2	0.9	21.7 ± 4.5	1.6	18.7 ± 0.6	0.8	114.3 ± 10.1	1.0	30.7 ± 14.5	0.6
Standard mutagen^Ψ^	103.3 ± 5.5	5.8^∗^	161.7 ± 8.0	12.1^∗^	376.3 ± 6.0	16.6^∗^	1035.7 ± 53.6	9.2^∗^	143.7 ± 10.3	3.0^∗^

*Note:* Each value represents the mean of revertant colonies ± SD, *n = *3. Bacterial mutagenicity analysis of Renogrit showing the average number of revertants per plate in *S. typhimurium* TA 1535, TA 1537, TA 98, and TA 100 and *E. coli uvrA* strains in the presence of metabolic activation (+S9). MR, mutagenicity ratio = number of revertant colonies in Renogrit or mutagen/number of revertant colonies in vehicle (DMSO).

Abbreviation: SD, standard deviation.

^Ψ^Standard mutagen: 2‐aminoanthracene (20 μg/plate) in TA 98, TA 100, TA 1535, and TA 1537 and *E. coli uvrA*.

^∗^denotes significant (*p* < 0.05) compared to vehicle (DMSO).

^#^ denotes mean of revertant colonies with standard deviation (SD).

### 3.2. Subacute Toxicity

#### 3.2.1. Effect of Renogrit on Body Weight and Feed Consumption

As presented in Figures 1(a) and 1(b), no statistically significant differences in body weight were observed between the Renogrit‐treated groups (100, 300, and 1000 mg/kg/day) and the control group at any time point during the study. Body weights remained comparable across all groups in both male Figure 1(a) and female rats Figure 1(b) throughout the treatment and recovery phases, indicating that Renogrit administration did not affect body weight under the conditions of this study. Similarly, no significant changes in body weight gain were noticed between the vehicle and Renogrit‐treated groups, both in the main and recovery arms, in male and female animals (Supporting Table 1).

FIGURE 1Body weight and feed consumption data (mean ± SD, *n* = 5 animals per sex): body weights (a & b) of animals from each experimental group. Vehicle control (G1), Renogrit, 100 mg/kg/day (G2), Renogrit, 300 mg/kg/day (G3), Renogrit, 1000 mg/kg/day (G4), vehicle control recovery (G1‐R), and Renogrit, 1000 mg/kg/day recovery (G4‐R)—were recorded individually on Days 1, 8, 15, 22, and 28. For recovery groups (G1‐R and G4‐R), additional measurements were taken on Days 35 and 42. Weekly feed consumption (c & d) was recorded weekly once for all groups throughout the study period and expressed total amount of feed consumption per animal per week.

**Figure:**
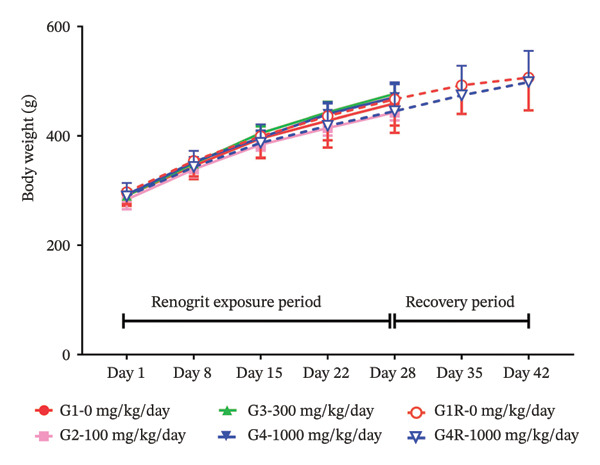
(a)

**Figure:**
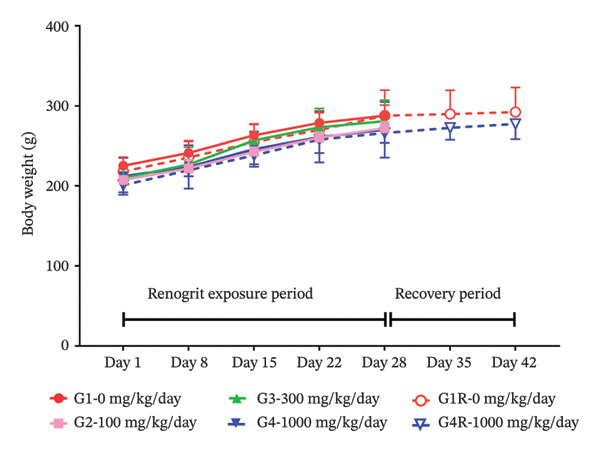
(b)

**Figure:**
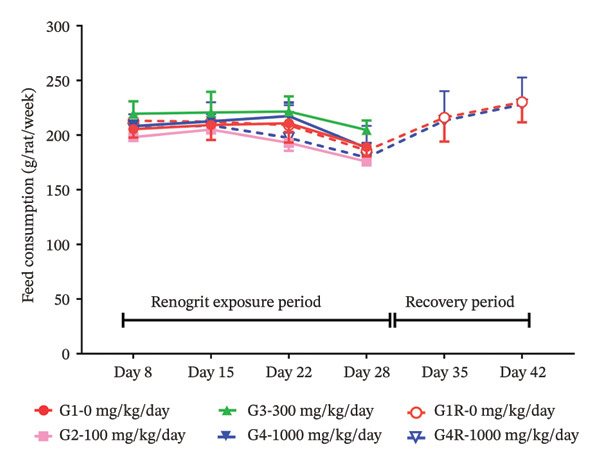
(c)

**Figure:**
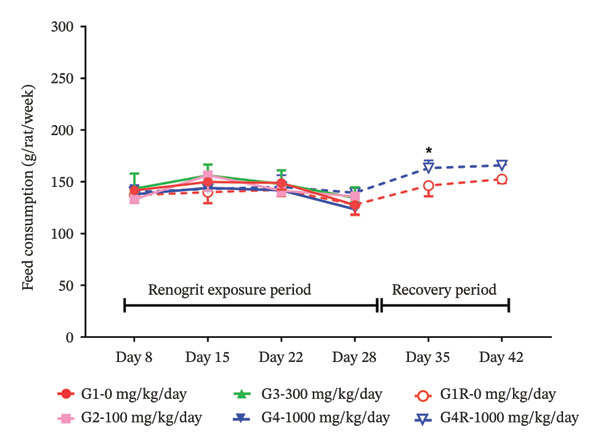
(d)

To further evaluate the potential effects of Renogrit on feed intake, weekly feed consumption was measured throughout the drug exposure and recovery periods. As shown in Figure 1(c) (male rats) and Figure 1(d) (female rats), there were no significant differences in feed consumption among the four groups (control and Renogrit‐treated groups at 100, 300, and 1000 mg/kg/day) during the majority of the study period. However, a statistically significant increase in feed consumption was observed in female animals on Day 35 in the high‐dose recovery group (1000 mg/kg/day) compared to the corresponding recovery control group (Figure 1(d)). This finding may suggest a transient, sex‐specific effect on appetite at the high dose during the recovery phase. The statistically significant increase in feed consumption detected in female rats during the recovery phase of the study is considered toxicologically inconsequential. This change was transient, noted only during the recovery period, and was not associated with similar findings during the dosing phase. Further, it was not accompanied by corresponding changes in body weight, clinical signs, or any related biochemical or pathological alterations. In our view, the observed effect might have resulted from post‐treatment recovery and may reflect compensatory or adaptive physiological responses. Therefore, the observed increase in feed consumption is regarded as a nonadverse, unrelated finding with no toxicological relevance to the test item.

#### 3.2.2. General and Detailed Clinical Sign Observations

To assess the potential toxicological effects of Renogrit, general clinical signs were monitored daily, and detailed clinical examinations [32] were conducted once weekly throughout the treatment and recovery periods. No abnormal clinical signs, morbidity, or mortality was observed in any of the groups (control and Renogrit‐treated groups at 100, 300, and 1000 mg/kg/day) in either male or female animals (Supporting Table 2). All experimental animals appeared clinically normal during the detailed weekly examinations across the entire study duration, indicating no overt signs of toxicity associated with Renogrit administration (Supporting Table 3).

#### 3.2.3. FOB

Neurological and behavioral assessments were done according to the OECD Test Guideline 407 [25]. The effect of Renogrit on motor activity, a key function regulated by the central nervous system, was evaluated by measuring total movements Figure (2a) and the number of rearings Figure (2b). The study outcomes did not detect any significant differences between Renogrit‐treated and vehicle control groups in both male and female animals during the main treatment and recovery periods. Accordingly, the observed findings imply that Renogrit did not adversely impact the central nervous system activity. Behavioral responses (Supporting Table 4), assessed through the FOB, were normal across all male and female subjects in both study phases. Neurological examinations (Supporting Table 5) revealed no abnormalities in muscle tone, gait, tail pinch response, olfactory response, eye blink response, or air‐righting reflex in either the main or recovery groups. Furthermore, convulsions, tremors, and stereotypic movements were absent in all animals. Landing foot splay distance measured at Weeks 4 and 6 showed no significant differences between Renogrit‐treated and control groups for both sexes during the main and recovery phases Figure (2c). The landing foot splay test is employed to study the effect of test articles on the neuromuscular function and motor coordination. The findings of this study demonstrated that Renogrit is devoid of any adverse effects on these physiological parameters. Additionally, autonomic responses (Supporting Table 6) were within normal limits in all animals throughout the FOB assessments, with no abnormalities detected in either main or recovery group.

FIGURE 2Functional observational battery (FOB) data (mean ± SD, *n* = 5 animals per sex): Motor activity and neurologic response are shown: number of movement (a), rearing (b), and landing foot splay (c) data from each experimental group—vehicle, Renogrit, 100, 300, and 1000 mg/kg/day, vehicle control recovery (R), and Renogrit 1000 mg/kg/day recovery group were recorded individually on Renogrit exposure period, i.e., Week 4. For recovery groups, additional measurements were taken in Week 6.

**Figure:**
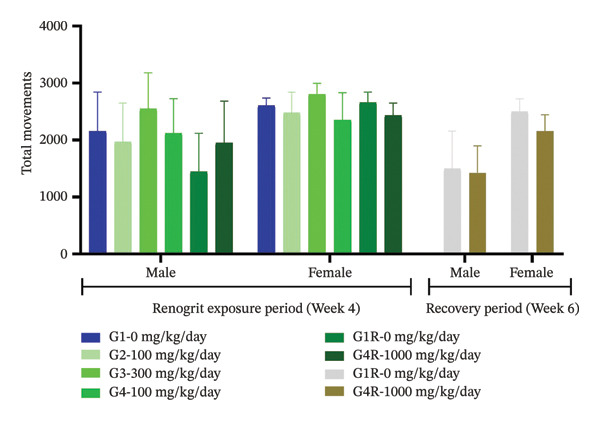
(a)

**Figure:**
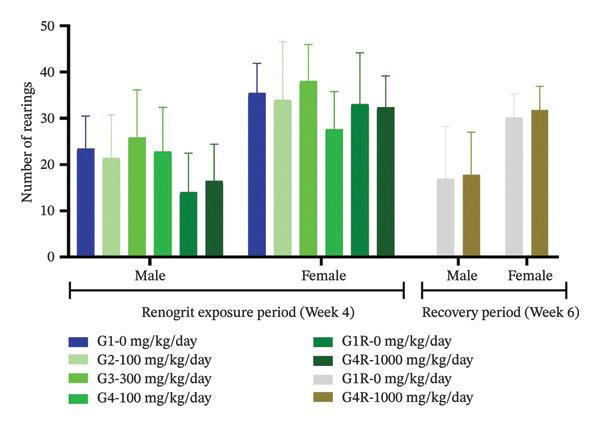
(b)

**Figure:**
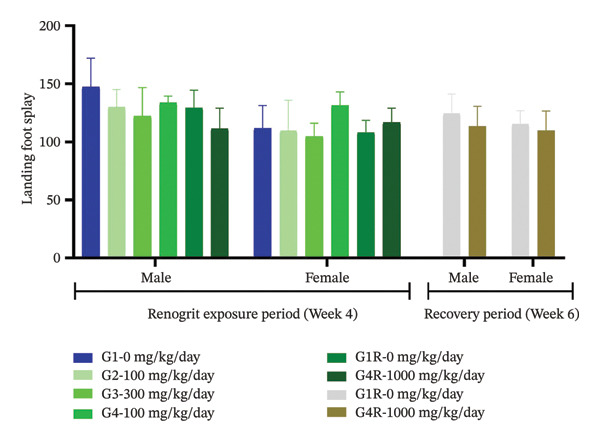
(c)

#### 3.2.4. Ophthalmological Observations

Renogrit administration for 28 consecutive days did not lead to any ocular abnormality in both male and female animals, when examined during Week 4, in both main and recovery arms (Supporting Table 7), as compared to the control groups (G1 and G1R, respectively). Furthermore, during Week 6, ophthalmoscopic examination of the eyes of both male and female rats, allocated to the recovery arms, did not reveal any delayed onset of aberrant ocular findings, when compared to the vehicle‐administered control group (Supporting Table 7).

#### 3.2.5. Effect of Renogrit on Hematological and Coagulation Parameters

The terminal hematological and coagulation parameters from rats allocated to both the main and recovery arms are summarized in Table 6. Compared to the control group, no significant differences were observed in most hematologic parameters across the Renogrit‐treated groups. However, male animals in the high‐dose group of the main study arm exhibited a significant increase in percentage of eosinophils (*p* < 0.01) relative to controls. In female animals assigned to the mean, a significant decrease in mean eosinophil percentage (*p* < 0.05) was noted in both the low‐dose (G2) and high‐dose (G4) groups. All other hematological parameters remained comparable to their respective controls. The observed alterations in eosinophil counts are likely to be incidental, given the absence of a clear dose–response relationship. Moreover, these observed values were within the historical reference ranges for SD rats at the site of study.

**TABLE 6:** Effect of Renogrit on hematological and coagulation parameters.

Parameter	28‐day Renogrit treatment (mg/kg/day)				14‐day recovery (mg/kg/day)	
	G1 (0)	G2 (100)	G3 (300)	G4 (1000)	G1R (0)	G4R (1000)
*Males (n = 5)*						
RBC (10^6^/μL)	7.49 ± 0.17	7.45 ± 0.17	7.35 ± 0.29	7.27 ± 0.38	8.14 ± 0.39	8.05 ± 0.14
HCT (%)	41.44 ± 0.96	41.10 ± 0.88	39.98 ± 1.14	40.64 ± 1.26	41.98 ± 1.19	42.74 ± 1.69
MCV (fL)	55.30 ± 0.91	55.14 ± 1.05	54.40 ± 1.09	55.94 ± 2.15	51.62 ± 1.26	53.12 ± 2.17
HGB (g/dL)	15.20 ± 0.45	14.98 ± 0.28	14.64 ± 0.44	14.84 ± 0.33	15.38 ± 0.59	15.46 ± 0.53
MCH (pg)	20.26 ± 0.40	20.12 ± 0.23	19.94 ± 0.34	20.42 ± 0.82	18.94 ± 0.55	19.22 ± 0.68
MCHC (g/dL)	36.60 ± 0.27	36.48 ± 0.40	36.68 ± 0.53	36.50 ± 0.46	36.62 ± 0.60	36.20 ± 0.51
PLT (10^3^/μL)	896.80 ± 53.56	943.80 ± 117.65	934.00 ± 71.08	890.80 ± 85.61	841.20 ± 128.66	860.20 ± 89.83
WBC (10^3^/μL)	12.60 ± 2.66	12.42 ± 4.82	12.11 ± 3.08	9.67 ± 2.57	11.54 ± 2.87	11.16 ± 4.07
NEU (%)	19.72 ± 8.74	24.64 ± 16.44	17.72 ± 4.13	20.54 ± 5.38	18.68 ± 4.85	14.54 ± 2.67
LYM (%)	72.70 ± 9.60	68.62 ± 16.73	75.52 ± 4.26	71.56 ± 4.91	72.34 ± 5.71	76.98 ± 4.77
MONO (%)	5.94 ± 0.56	4.84 ± 1.14	5.06 ± 1.12	6.00 ± 1.47	6.44 ± 1.47	6.48 ± 1.96
EOS (%)	0.86 ± 0.18	0.94 ± 0.11	0.74 ± 0.15	1.20 ± 0.10^∗∗^	1.36 ± 0.55	1.18 ± 0.43
BASO (%)	0.78 ± 0.31	0.96 ± 0.43	0.96 ± 0.38	0.70 ± 0.10	1.18 ± 0.29	0.82 ± 0.33
RETI (%)	1.34 ± 0.44	1.30 ± 0.27	1.14 ± 0.45	1.28 ± 0.37	1.16 ± 0.18	1.00 ± 0.10
PT (Sec.)	16.06 ± 1.28	14.20 ± 2.26	17.64 ± 2.05	16.88 ± 1.54	22.82 ± 1.66	19.84 ± 2.35^#^
aPTT (Sec.)	22.58 ± 0.86	25.20 ± 3.12	23.48 ± 3.46	25.42 ± 2.85	16.80 ± 1.59	17.06 ± 1.01

*Females (n = 5)*						
RBC (10^6^/μL)	6.95 ± 0.29	6.92 ± 0.24	7.18 ± 0.42	7.16 ± 0.20	7.63 ± 0.67	7.44 ± 0.10
HCT (%)	38.26 ± 1.63	38.16 ± 1.15	38.98 ± 2.17	37.80 ± 1.50	41.20 ± 3.42	40.32 ± 1.24
MCV (fL)	55.02 ± 0.91	55.14 ± 1.30	54.30 ± 1.71	52.82 ± 1.68	54.02 ± 1.58	54.20 ± 0.96
HGB (g/dL)	14.36 ± 0.59	14.46 ± 0.15	14.64 ± 0.75	14.34 ± 0.44	15.26 ± 1.36	14.90 ± 0.54
MCH (pg)	20.66 ± 0.47	20.90 ± 0.55	20.42 ± 0.54	20.00 ± 0.55	19.98 ± 0.75	20.04 ± 0.49
MCHC (g/dL)	37.50 ± 0.60	37.92 ± 0.80	37.64 ± 0.38	37.90 ± 0.34	37.00 ± 0.40	37.02 ± 0.28
PLT (10^3^/μL)	850.40 ± 69.07	914.80 ± 104.49	878.00 ± 117.04	941.40 ± 90.49	844.20 ± 78.21	807.40 ± 88.57
WBC (10^3^/μL)	8.90 ± 1.81	7.83 ± 2.73	10.49 ± 4.39	9.34 ± 2.51	9.37 ± 2.11	9.40 ± 1.75
NEU (%)	13.22 ± 3.35	14.12 ± 4.89	15.14 ± 5.50	19.40 ± 3.82	14.06 ± 2.82	12.24 ± 3.31
LYM (%)	80.62 ± 4.40	80.96 ± 5.89	79.30 ± 6.98	75.66 ± 4.28	80.46 ± 3.68	81.92 ± 4.18
MONO (%)	3.94 ± 1.05	3.34 ± 1.08	3.64 ± 1.35	3.30 ± 0.37	3.64 ± 1.11	3.76 ± 0.93
EOS (%)	1.62 ± 0.51	0.92 ± 0.08^∗^	1.40 ± 0.41	1.00 ± 0.20^∗^	1.06 ± 0.38	1.36 ± 0.40
BASO (%)	0.60 ± 0.14	0.66 ± 0.27	0.52 ± 0.16	0.64 ± 0.11	0.78 ± 0.18	0.72 ± 0.33
RETI (%)	1.06 ± 0.44	1.30 ± 0.38	1.18 ± 0.26	1.32 ± 0.19	1.32 ± 0.30	1.50 ± 0.22
PT (Sec.)	16.60 ± 1.35	16.54 ± 0.69	16.14 ± 1.63	15.42 ± 1.54	17.88 ± 1.26	18.46 ± 0.87
aPTT (Sec.)	23.24 ± 1.99	22.34 ± 2.95	24.08 ± 4.25	22.44 ± 2.22	15.22 ± 1.25	16.78 ± 1.89

*Note:* Data presented as mean ± standard deviation (*n* = 5). G1 (0): Vehicle control animals received 0.5% methyl cellulose (10 mL/kg/day); G2 (100), G3 (300), and G4 (1000): Experimental animals received Renogrit at corresponding doses of 100, 300, and 1000 mg/kg/day for 28 consecutive days. G1R (0): Vehicle recovery group animals, observed 14 days after withdrawing 28‐day 0.5% methyl cellulose treatment. G4R (1000): Renogrit recovery group animals, observed 14 days after withdrawing 28‐day Renogrit treatment. HCT, hematocrit; HGB, hemoglobin; PLT, platelet; NEU, neutrophils; LYM, lymphocytes; MONO, monocytes; EOS, eosinophils; BASO, basophils; RETI, reticulocyte.

Abbreviations: APTT, activated partial thromboplastin time; MCH, mean corpuscular hemoglobin; MCHC, mean corpuscular hemoglobin concentration; MCV, mean corpuscular volume; PT, prothrombin time; RBC, red blood cell count; WBC, white blood cell count.

^∗^ *p* < 0.05.

^∗∗^ *p* < 0.01 Vs. G1 (one‐way ANOVA followed by Dunnett’s multiple comparison test).

^#^ *p* < 0.05 vs. G1R (Student’s *t*‐test).

PT and aPTT values in both main and recovery groups treated with Renogrit were largely comparable to those of the respective control groups in both male and female animals. However, a statistically significant decrease (*p* < 0.05) in PT was observed in male animals from the high‐dose recovery group (1000 mg/kg/day) compared to the control recovery group (G1R). These findings indicate sporadic findings, which are within the historical reference ranges of SD rats at the site of the study.

#### 3.2.6. Effect of Renogrit on Clinical Chemistry Parameters

The terminal clinical chemistry parameters from animals assigned to both the main and recovery arms are summarized in Table 7. Largely, no significant differences were observed in the clinical chemistry parameters during either the drug exposure or recovery periods across all Renogrit‐treated groups, when compared to their respective control groups. Nevertheless, biochemical analysis in rats from the recovery arm revealed a significant decrease (*p* < 0.05) in PRO levels in male animals treated with Renogrit at 1000 mg/kg/day, compared to its respective control group; however, this observed change remained within normal physiological limits and the historical ranges for SD rats at the study site. Moreover, female animals assigned to the high‐dose recovery group exhibited a significant decrease (*p* < 0.05) in serum cholesterol levels compared to the control recovery group. This finding is also unlikely to be toxicologically relevant as the values were well within the historical reference ranges for female SD rats at the study site.

**TABLE 7:** Effect of Renogrit on clinical chemistry parameters.

Parameter	28‐day Renogrit treatment (mg/kg/day)				14‐day recovery (mg/kg/day)	
	G1 (0)	G2 (100)	G3 (300)	G4 (1000)	G1R (0)	G4R (1000)
*Males (n = 5)*						
AST (U/L)	146.58 ± 41.82	132.48 ± 31.35	160.34 ± 70.21	113.02 ± 16.32	129.80 ± 21.71	108.30 ± 21.96
ALT (U/L)	49.04 ± 11.17	52.90 ± 11.16	63.06 ± 29.35	46.64 ± 5.93	57.58 ± 5.30	57.90 ± 6.10
ALP (U/L)	237.00 ± 40.31	222.00 ± 22.33	260.20 ± 16.48	281.20 ± 42.52	234.60 ± 57.47	214.00 ± 30.87
BILT (mg/dL	0.07 ± 0.01	0.05 ± 0.03	0.07 ± 0.01	0.06 ± 0.01	0.08 ± 0.02	0.10 ± 0.01
BUL (mg/dL)	37.70 ± 4.73	33.26 ± 1.72	35.90 ± 2.70	36.16 ± 3.60	32.50 ± 3.08	34.68 ± 2.82
CBUN (mg/dL)	17.62 ± 2.21	15.54 ± 0.80	16.78 ± 1.26	16.90 ± 1.68	15.19 ± 1.44	16.20 ± 1.32
CREAT (mg/dL)	0.09 ± 0.04	0.09 ± 0.04	0.09 ± 0.05	0.12 ± 0.04	0.13 ± 0.05	0.12 ± 0.08
GLU (mg/dL)	137.42 ± 26.38	120.42 ± 17.42	131.96 ± 8.19	133.50 ± 14.08	161.04 ± 22.20	149.88 ± 26.69
CHOL (mg/dL)	72.40 ± 10.38	70.20 ± 9.28	64.80 ± 12.48	69.20 ± 15.32	79.40 ± 14.98	74.20 ± 8.47
PRO (g/dL)	7.51 ± 0.33	7.20 ± 0.36	7.60 ± 0.29	7.52 ± 0.37	7.94 ± 0.32	7.52 ± 0.20^#^
ALB (mg/dL)	2.81 ± 0.18	2.71 ± 0.12	2.86 ± 0.05	2.83 ± 0.18	2.95 ± 0.08	2.88 ± 0.08
PAR	2.67 ± 0.08	2.65 ± 0.07	2.66 ± 0.11	2.66 ± 0.07	2.69 ± 0.07	2.61 ± 0.05
Na+ (mmol/L)	159.97 ± 11.75	165.69 ± 7.41	169.12 ± 8.03	165.16 ± 10.33	166.05 ± 21.17	179.28 ± 11.91
K+ (mmol/L)	5.93 ± 0.31	5.44 ± 0.36	5.66 ± 0.72	6.29 ± 0.95	5.94 ± 0.91	5.61 ± 0.39

*Females (n = 5)*						
AST (U/L)	110.16 ± 7.94	112.22 ± 27.85	121.42 ± 23.30	120.14 ± 15.40	115.89 ± 17.28	116.12 ± 20.07
ALT (U/L)	39.78 ± 4.47	44.70 ± 4.57	46.46 ± 4.40	39.86 ± 6.26	45.70 ± 2.47	46.88 ± 7.62
ALP (U/L)	126.80 ± 37.89	154.60 ± 14.81	172.20 ± 49.60	146.00 ± 32.03	147.20 ± 33.61	128.20 ± 19.38
BILT (mg/dL	0.08 ± 0.02	0.08 ± 0.02	0.08 ± 0.01	0.09 ± 0.01	0.10 ± 0.02	0.11 ± 0.02
BUL (mg/dL)	35.70 ± 3.12	36.50 ± 6.72	36.64 ± 5.59	39.54 ± 2.06	39.90 ± 5.93	42.60 ± 2.61
CBUN (mg/dL)	16.88 ± 1.46	17.06 ± 3.14	17.12 ± 2.61	18.48 ± 0.97	18.64 ± 2.77	19.91 ± 1.22
CREAT (mg/dL)	0.17 ± 0.05	0.17 ± 0.06	0.18 ± 0.05	0.18 ± 0.04	0.10 ± 0.03	0.13 ± 0.04
GLU (mg/dL)	154.32 ± 59.20	161.08 ± 69.89	137.44 ± 43.01	121.86 ± 37.72	145.92 ± 38.28	118.62 ± 20.54
CHOL (mg/dL)	86.20 ± 12.60	82.20 ± 12.19	93.80 ± 13.92	72.40 ± 15.47	102.40 ± 16.38	75.20 ± 19.07^#^
PRO (g/dL)	7.53 ± 0.39	8.11 ± 0.55	7.68 ± 0.52	7.74 ± 0.60	8.41 ± 0.47	7.86 ± 0.43
ALB (mg/dL)	3.04 ± 0.09	3.27 ± 0.33	3.26 ± 0.14	3.20 ± 0.27	3.41 ± 0.22	3.16 ± 0.17
PAR	2.48 ± 0.07	2.49 ± 0.10	2.36 ± 0.16	2.42 ± 0.07	2.46 ± 0.07	1.98 ± 1.09
Na+ (mmol/L)	175.50 ± 8.58	176.93 ± 9.94	170.59 ± 7.13	175.35 ± 15.91	175.56 ± 6.46	178.66 ± 11.14
K+ (mmol/L)	5.47 ± 1.13	7.09 ± 1.71	5.31 ± 1.02	6.20 ± 2.62	6.60 ± 1.55	5.52 ± 0.36

*Note:* Data presented as mean ± standard deviation (*n* = 5). G1 (0): Vehicle control animals received 0.5% methyl cellulose (10 mL/kg/day); G2 (100), G3 (300), and G4 (1000): Experimental animals received Renogrit at corresponding doses of 100, 300, and 1000 mg/kg/day for 28 consecutive days. G1R (0): Vehicle recovery group animals, observed 14 days after withdrawing 28‐day 0.5% methyl cellulose treatment. G4R (1000): Renogrit recovery group animals, observed 14 days after withdrawing 28‐day Renogrit treatment. AST, aspartate aminotransferase; ALT, alanine aminotransferase; ALP, alkaline phosphatase; CREAT, creatinine; BILT, total bilirubin; GLU, glucose; CHOL, total cholesterol; PRO, total protein; ALB, albumin; Na, sodium; and K, potassium.

Abbreviations: BUL, blood urea level; CBUN, calculated blood urea nitrogen; PAR, protein:albumin ratio.

^#^ *p* < 0.05 vs. G1R (Student’s *t*‐test).

#### 3.2.7. Macroscopic Observation and Organ Weights of Renogrit‐Administered Animals

When compared to their respective controls, gross pathological examinations of organs in male and female animals assigned to both main and recovery arms did not reveal any abnormalities across all treatment groups.

The relative organ weights of male and female rats w.r.t their fasting body weights, from all the six experimental groups, are presented in Table 8. In the main study arm, relative organ weights of Renogrit‐administered rats at all the tested doses were by and large comparable to the vehicle‐administered control group (G1). However, among the female rats, a statistically significant increase in the relative weight of kidney was evident in the group that received Renogrit at the low dose of 100 mg/kg/day (*p* < 0.05). Nevertheless, this observed finding is inferred to be incidental due to lack of dose‐dependency. Similarly, in the recovery arm, relative organ weights of organs excised from animals that received the high dose of Renogrit (1000 mg/kg/day) were largely comparable to the control group. Nonetheless, compared to the recovery control group (G1R), a statistically significant (*p* < 0.05) increase in relative liver weight was noted in female rats that received Renogrit. However, the observed effect is unlikely to be of toxicological relevance as the relative liver weight was within the historical reference range for female SD rats at the site of the study.

**TABLE 8:** Effect of Renogrit on relative organ weights represented as percentage of terminal body weights.

Parameter	28‐day Renogrit treatment (mg/kg/day)				14‐day recovery (mg/kg/day)	
	G1 (0)	G2 (100)	G3 (300)	G4 (1000)	G1R (0)	G4R (1000)
*Males (n = 5)*						
Fasting body weight (g)	441.89 ± 52.89	421.01 ± 12.88	456.18 ± 21.41	449.70 ± 21.29	497.09 ± 55.12	477.92 ± 53.87
Liver	3.688 ± 0.225	3.618 ± 0.512	4.059 ± 0.340	3.797 ± 0.445	4.079 ± 0.512	3.747 ± 0.143
Kidneys	0.726 ± 0.059	0.710 ± 0.056	0.747 ± 0.030	0.744 ± 0.046	0.763 ± 0.053	0.756 ± 0.049
Adrenals	0.016 ± 0.001	0.013 ± 0.005	0.013 ± 0.003	0.016 ± 0.003	0.017 ± 0.002	0.015 ± 0.003
Spleen	0.181 ± 0.012	0.186 ± 0.035	0.183 ± 0.016	0.179 ± 0.025	0.158 ± 0.018	0.182 ± 0.024
Brain	0.493 ± 0.046	0.491 ± 0.031	0.450 ± 0.033	0.453 ± 0.021	0.429 ± 0.058	0.453 ± 0.034
Heart	0.319 ± 0.019	0.330 ± 0.017	0.328 ± 0.023	0.266 ± 0.149	0.348 ± 0.067	0.324 ± 0.031
Thymus	0.122 ± 0.031	0.131 ± 0.021	0.130 ± 0.029	0.111 ± 0.035	0.080 ± 0.026	0.088 ± 0.029
Testes	0.743 ± 0.095	0.809 ± 0.037	0.739 ± 0.045	0.755 ± 0.065	0.711 ± 0.065	0.719 ± 0.104
Epididymis	0.260 ± 0.029	0.275 ± 0.016	0.266 ± 0.031	0.254 ± 0.020	0.267 ± 0.023	0.286 ± 0.037
Prostate + seminal vesicle	0.712 ± 0.024	0.626 ± 0.027	0.626 ± 0.073	0.631 ± 0.071	0.764 ± 0.079	0.757 ± 0.060

*Females (n = 5)*						
Fasting body weight (g)	275.03 ± 14.91	262.50 ± 5.29	271.90 ± 20.32	259.82 ± 29.84	284.51 ± 28.14	269.04 ± 16.82
Liver	3.758 ± 0.206	3.704 ± 0.289	3.654 ± 0.497	3.646 ± 0.300	3.547 ± 0.144	3.784 ± 0.153^#^
Kidneys	0.688 ± 0.058	0.808 ± 0.084^∗^	0.739 ± 0.036	0.741 ± 0.057	0.709 ± 0.045	0.725 ± 0.037
Adrenals	0.028 ± 0.003	0.030 ± 0.005	0.029 ± 0.005	0.032 ± 0.004	0.029 ± 0.004	0.029 ± 0.003
Spleen	0.195 ± 0.024	0.206 ± 0.053	0.190 ± 0.037	0.205 ± 0.027	0.206 ± 0.031	0.197 ± 0.014
Brain	0.737 ± 0.022	0.750 ± 0.038	0.741 ± 0.065	0.785 ± 0.055	0.727 ± 0.070	0.731 ± 0.037
Heart	0.347 ± 0.041	0.367 ± 0.026	0.337 ± 0.017	0.354 ± 0.031	0.354 ± 0.026	0.353 ± 0.020
Thymus	0.170 ± 0.018	0.153 ± 0.052	0.179 ± 0.061	0.156 ± 0.042	0.141 ± 0.019	0.165 ± 0.030
Ovaries	0.054 ± 0.009	0.057 ± 0.008	0.047 ± 0.005	0.060 ± 0.006	0.048 ± 0.012	0.060 ± 0.003
Uterus + cervix	0.250 ± 0.078	0.348 ± 0.056	0.291 ± 0.073	0.344 ± 0.083	0.329 ± 0.114	0.338 ± 0.079

*Note:* Data presented as mean ± standard deviation (*n* = 5). G1 (0): Vehicle control animals received 0.5% methyl cellulose (10 mL/kg/day); G2 (100), G3 (300), and G4 (1000): Experimental animals received Renogrit at corresponding doses of 100, 300, and 1000 mg/kg/day for 28 consecutive days. G1R (0): Vehicle recovery group animals, observed 14 days after withdrawing 28‐day 0.5% methyl cellulose treatment. G4R (1000): Renogrit recovery group animals, observed 14 days after withdrawing 28‐day Renogrit treatment.

^∗^ *p* < 0.05 vs. G1 (one‐way ANOVA followed by Dunnett’s multiple comparison test).

^#^ *p* < 0.05 vs. G1R (Student’s *t*‐test).

Furthermore, the absolute organ weights and the relative organ weights w.r.t brain weight from the experimental groups are presented in Supporting Tables 8 and 9, respectively. Analysis of both these parameters revealed that the absolute and relative organ weights (w.r.t. brain weight) of Renogrit‐administered rats, allocated to both the main and recovery groups, were comparable to those of their corresponding controls, respectively.

#### 3.2.8. Histopathological Observations

Histological findings detected in the animals from the main study arms that received vehicle and the high dose of Renogrit are tabulated in Table 9, and the representative photomicrographs of various organs are depicted in Figures 3(a) and 3(b). Oral administration of high dose of Renogrit to both male and female SD rats for 28 consecutive days did not induce any treatment‐related local or systemic toxicologically relevant adverse effects in any organs or tissues. Central vein (CV) and hepatocyte plates (HP) were intact in the liver tissue specimens of both control and Renogrit. Alveolar space (AVS), no fibrotic debris, clear air space (AS), and no inflammatory infiltrates to indicate healthy normal lungs were observed in normal and Renogrit‐treated lung tissue specimens. The sections of normal and Renogrit‐treated kidney show distinct cortex and medulla. The cortex contains numerous renal corpuscles composed of glomeruli (GLO) surrounded by Bowman’s capsule. Proximal and distal convoluted tubules are seen with normal tubular epithelial lining (TE). No evidence of necrosis, congestion, or inflammatory infiltration was observed. The intercalated disk (ID), well‐organized myocardial fibers (MF), central nuclei, intact endocardium and epicardium, and the absence of inflammatory cells, necrosis, fibrosis, or vascular abnormalities were seen in the heart tissue sections of both normal and Renogrit‐treated rats to demonstrate normal histology. Normal and Renogrit‐treated rat cerebrum part of the brain shows normal findings, such as well‐organized cortical layering, intact neuropil (N), normal glial cell distribution, intact neurons with vesicular nuclei, and prominent nucleoli in addition to the absence of inflammation, necrosis, edema, or vascular abnormalities. Intact ductal epithelium (DE) was seen in all the specimens of pancreas to show normal cellular architecture of both normal and Renogrit‐treated rats. Normal distribution of acidophils (ACI), basophils, and chromophobes in the anterior lobe of pituitary glands was seen in normal and Renogrit‐treated specimens. Rich vascularization (V) and intact connective tissue are seen in specimens of normal and Renogrit‐treated rats’ adrenal gland (Figure 3(a)).

**TABLE 9:** Histopathological investigation of animals treated with vehicle and high dose of Renogrit.

Organ and finding	Grade and severity	Males (*N* = 5)		Females (*N* = 5)	
		G1 (0)	G4 (1000)	G1 (0)	G4 (1000)
Adrenal gland	Multifocal–minimal	1/5	0/5	0/5	0/5
Vacuolation					

Liver	Focal–mild	1/5	0/5	0/5	0/5
Extramedullary hematopoiesis					

Kidney	Multifocal–mild	0/5	0/5	0/5	1/5
Tubular dilatation					

*Note:* Data are presented as number of animals with respective findings out of the total number of animals. G1 (0): Vehicle control animals received 0.5% methyl cellulose (10 mL/kg/day); G4 (1000): Experimental animals received Renogrit at 1000 mg/kg/day for 28 consecutive days.

FIGURE 3(a) Representative histological photomicrographs of liver, lung, kidney, heart, cerebrum, pancreas, pituitary gland, and adrenal gland from male and female rats. CV, central vein; HP, hepatocyte plates; AVS, alveolar space; AS, air space; GLO, glomerulus; TE, tubular epithelium; ID, intercalated disk; MF, myocardial fibers; N, neuropil; DE, ductal epithelium; ACI, acidophils; V, vascularization. (b) Representative histological photomicrographs of skin, bone marrow, spleen, urinary bladder, colon, testis, prostate, seminal vesicles, ovary, mammary gland, and uterus. SG, sebaceous glands; M, megakaryocytes; WP, white pulp; RP, red pulp; ET, epithelium; M, mucosa; SM, submucosa; F, follicles; ST, seminiferous tubule; EL, epithelial lining; D, duct; MF, mucosal fold; LE, luminal epithelium. Tissue sections were stained with hematoxylin and eosin (H&E) and examined microscopically. Magnification: × 100. G1 (0); vehicle control animals received 0.5% methyl cellulose orally (10 mL/kg/day), G4 (1000): experimental animals received Renogrit orally at 1000 mg/kg/day for 28 consecutive days.

**Figure:**
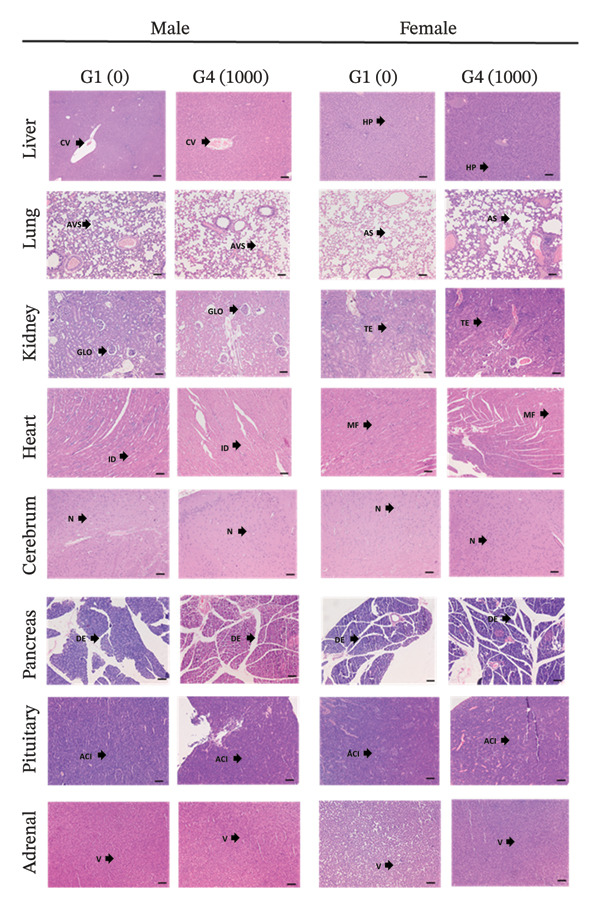
(a)

**Figure:**
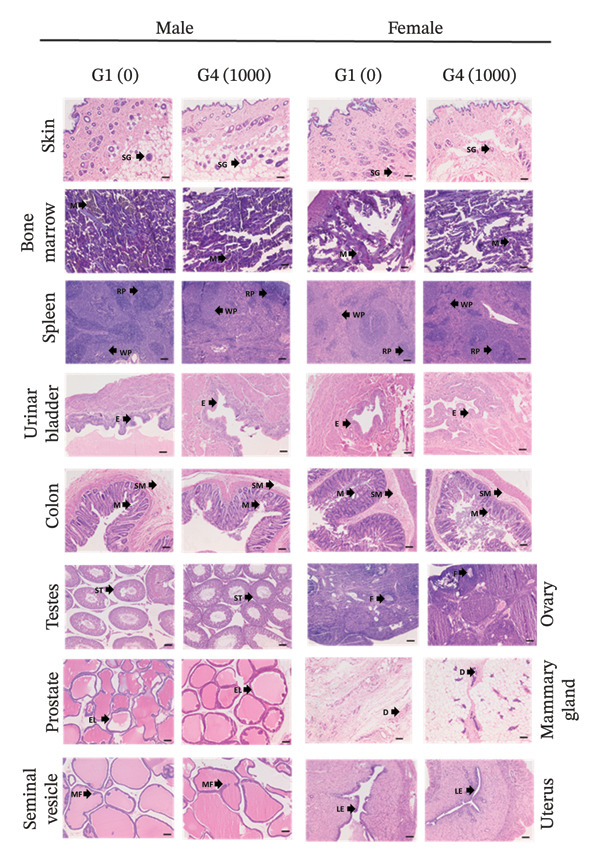
(b)

Normal hair follicles and sebaceous glands (SG), intact keratinized epidermis, the absence of inflammatory infiltrate, and structural abnormalities were seen in the histology of skin of both normal and Renogrit‐treated specimens. Bone marrow smear of both normal and Renogrit‐treated rats showed adequate number of megakaryocytes (M), intact stromal and vascular components, active hematopoiesis, and the absence of cellular atypia, necrosis, or fibrosis. Histological investigation of both normal control and Renogrit‐treated spleen tissue revealed intact capsule and trabeculae, distinction between white pulp (WP) and red pulp (RP), normal splenic cords and sinusoids, normal lymphoid follicles, and the absence of necrosis, congestion, hemorrhage, inflammation, or lymphoid depletion. Uniform epithelial thickness (ET), intact urothelial surface, the presence of normal umbrella cells, no ulceration or erosion, no edema or hemorrhage, and no inflammatory cell infiltration were observed in urinary bladder tissues of the rats treated with vehicle and Renogrit. Colon histology of rats treated with vehicle and Renogrit shows intact mucosa (M) and submucosa (SM), parallel crypts, abundant goblet cells, intact epithelial surface, minimal inflammatory cells, and no crypt distortion or ulceration. The histology of ovaries of animals treated with vehicle and Renogrit showed follicles (F) at various developmental stages, corpus luteum depending on cycle stage, no inflammatory infiltrate, and no fibrosis, necrosis, or neoplasia to indicate no abnormalities upon Renogrit treatment. Complete and active spermatogenesis stages found that normal Sertoli cell morphology, normal Leydig cell distribution, uniform seminiferous tubule (ST) diameter, and no degeneration, necrosis, no tubular atrophy, or fibrosis were examined in all the specimens of rat testis treated with vehicle and Renogrit. Well‐organized acini with uniform epithelial lining (EL), cuboidal to columnar secretory cells, thin smooth muscle layer around acini, minimal connective tissue, no fibrosis, no inflammatory cells, necrosis, or atypia were found in all the tissue specimens of prostate to show no abnormality in prostate tissue upon Renogrit treatment. Intact ductal–alveolar (D) architecture, lumen containing secretory material, no inflammatory infiltrate, fibrosis, or hyperplasia, and uniform epithelial nuclei were present in all the examined specimens of rats, belonging to normal control and Renogrit‐treated rats to indicate no abnormality. The uterus of female rats treated with Renogrit showed intact luminal epithelium (LE), uniform glandular architecture in endometrium, endometrial stroma without edema or inflammatory infiltrate, well‐organized myometrial smooth muscle bundles, normal vasculature and connective tissue, no hyperplasia, metaplasia, or necrosis which is similar to the normal control rats. High mucosal fold (MF), eosinophilic luminal secretions, thick smooth muscle wall, and pseudostratified columnar epithelium are seen in normal and Renogrit‐treated specimens, which are the normal histoarchitecture of the seminal vesicle (Figure 3(b)). Thus, all examined tissue specimens retained normal cellular architecture, with no histopathological abnormalities observed following Renogrit administration. A few incidental histopathological findings were noted in both vehicle control and Renogrit‐administered animals (Table 9). However, these observations were considered spontaneous, incidental, or congenital in nature and not related to Renogrit administration. Furthermore, such histopathological changes are commonly reported as background lesions in laboratory rats according to the existing literature. The observation of minimal, multifocal vacuolation in the adrenal gland (Table 9) is typically localized to the cortical regions (particularly the zona fasciculata) and is often associated with physiological lipid accumulation related to normal steroidogenesis. The observation was noted in one male animal from the normal control group and is therefore considered an incidental finding [33].

Further, mild, focal extramedullary hematopoiesis was observed in the liver from a male animal allocated to the vehicle‐treated normal control group (Table 9). It has been reported that liver under certain circumstances may be a site for extramedullary hematopoiesis, microscopically evident as a heterogeneous mass. In this study, the minimal to mild severity of this finding in the concerned animal, with a focal distribution and absence of associated hepatocellular injury or inflammation, supports its interpretation as an incidental finding [34]. Furthermore, mild tubular dilatation was observed in the kidneys of one female animal administered with high dose of Renogrit (Table 9). It has been previously reported that, in rodents, a few dilated tubules in the kidney may be regarded as normal histologic variation. The low severity of the histological finding observed in the animal, along with the absence of accompanying degenerative changes, such as interstitial inflammation, fibrosis, or obstructive lesions, substantiates this interpretation as an incidental finding and not related to the test article [35].

## 4. Discussion

The individual phytoconstituents of Renogrit have been traditionally prescribed in Indian systems of medicine to support the healthy functioning of the kidneys, by virtue of their anti‐inflammatory, antioxidant, lithotriptic, diuretic, and antibacterial properties. More recently, studies conducted at our laboratory have demonstrated pharmacological effects of Renogrit in mitigating vancomycin‐ and cisplatin‐induced nephrotoxicity in both in vitro and in vivo models [22, 23], respectively. These preclinical study outcomes thereby suggest that Renogrit possesses potential to assuage renal damage induced by chemotherapeutic drugs. Consequently, Renogrit is a prospective candidate for detailed nonclinical and ultimately clinical investigations.

Nonclinical safety evaluation plays a pivotal role in establishing the safety profile of botanical drug–based medicines and is a prerequisite for their progression into clinical trials. Despite the growing interest in the therapeutic potential and diverse pharmacological activities of these medicines, their possible adverse effects are often underreported or insufficiently characterized. Therefore, rigorous genotoxicity and systematic toxicity assessments are essential to ensure the safe use of these natural products in both preclinical and clinical contexts [36]. Accordingly, in this study, we evaluated the mutagenic potential of Renogrit by testing it in a bacterial reverse mutation (Ames) test. In addition, we also assessed the nonclinical safety of Renogrit after repeatedly administering it to male and female SD rats orally for 28 successive days, which was followed by a 14‐day treatment‐free recovery period.

The mutagenic potential of Renogrit was tested as per the OECD Test Guideline 471 [24] using histidine‐dependent *S. typhimurium* tester strains, such as TA 98, TA 100, TA 1537, and TA 1535, and tryptophan‐dependent *E. coli* uvrA strain, in the presence and absence of metabolic activation with rat liver S9 fraction. As inferred from the computed MR, which was found lower than the cut‐off value of 2, in both the experimental conditions, Renogrit was adjudged to be nonmutagenic up to a concentration of 5 mg/plate. Further, in vivo nonclinical safety of Renogrit, evaluated in accordance with the OECD Test Guideline 407 [25], ascertained it to be safe up to the dose of 1000 mg/kg/day, in both male and female SD rats. Although certain parameters in Renogrit‐administered rats did demonstrate statistically significant changes when compared to the vehicle‐administered control animals, they were not judged to be toxicologically relevant and not directly attributed to Renogrit administration. This conclusion was arrived at because the observed alterations were either not dose‐dependent or were well within the historical reference ranges for SD rats at the study site. Accordingly, the NOAEL for Renogrit subsequent to repeated 28‐day oral administration was determined to be 1000 mg/kg/day. The previously ascertained preclinical renoprotective effects of Renogrit coupled with its nonmutagenicity and acceptable preclinical in vivo safety, established in this study, provide the foundation for its future nonclinical as well as clinical evaluation.

In the public domain, limited published scientific literature exists that cover the mutagenicity and in vivo subacute toxicity evaluation of the extracts of the individual botanical drugs present in Renogrit. Nevertheless, it is rather important to mention the reported safety evaluations. In this light, it is rather important to compute the doses of the individual botanical drugs present in Renogrit which the rats of different experimental groups would have received, in order to compare the findings of the previously published nonclinical safety studies with this study. Accordingly, based on their relative proportion in Renogrit tablet, the daily doses of the extracts of *Achyranthes aspera* L., *Saxifraga ligulata* Murray, *Butea frondosa* Roxb. ex Willd., *Crataeva nurvala* Buch.‐Ham., *Boerhavia diffusa* L., and *Tribulus terrestris* L., which the experimental animals would have received, could be calculated to be 13.2, 39.7, and 132.4 mg/kg/day. Similarly, the daily doses of *Cichorium intybus* L. whole plant extract, which the animals would have received, could be computed to be 9.8, 29.4, and 98 mg/kg/day. Further, in the case of *Cichorium intybus* L. seed extract, the daily dose received by the animals could be calculated to be 3.3, 10.0, and 33.3 mg/kg/day.

Of these phytoconstituents of Renogrit, the subacute toxicity of the aqueous extract of *Butea monosperma* (Lam.) (a synonym of *Butea frondosa* Roxb. ex Willd.) has been evaluated in mice, wherein the animals received the extract by oral route for 30 consecutive days at the doses of 250, 500, 1500, 3000, and 4500 mg/kg/day [37]. These administered doses are roughly equivalent to 125, 250, 750, 1500, and 2250 mg/kg in rats based on the differences in the body surface areas of mice and rats [38]. When compared to the control group, the experimental findings in the test article–administered animals revealed no significant changes in percentage body weight gain, hematological parameters, and hepatic and kidney function markers up to the dose of 4500 mg/kg/day. Similarly, the histological architectures of kidney, liver, heart, and brain in the test article–administered animals were comparable to the control group. In our study, even the highest dose of the extract of *Butea frondosa* Roxb. ex Willd. that the rats would have received (132.4 mg/kg/day) is comparable to the lowest tested dose of the test article in the reported study and only a fraction of the NOAEL.

The subacute toxicity of the hydroalcoholic extract of *Boerhavia diffusa* L. roots, also a constituent of Renogrit, has been assessed in Wistar rats subsequent to oral administration of the extract at a single dose of 1000 mg/kg/day for 28 consecutive days [14]. In this study, no toxicologically relevant effects associated with the extract were observed, thereby confirming its nonclinical safety subsequent to subacute administration of the tested dose. In our study, the highest dose of *Boerhavia diffusa* root extract, which the rats would have received (132.4 mg/kg/day), is again a fraction of its previously reported NOAEL, i.e., 1000 mg/kg/day.

Another botanical drug present in Renogrit, namely *Cichorium intybus* L., has been investigated for its mutagenic potential using the Ames test as well as its subacute toxicity assessment in SD rats of both genders [39]. In this published study, the ethanolic root extracts of the plant were evaluated. The OECD‐driven Ames test outcomes concluded that the root extract was nonmutagenic up to the concentration of 5 mg/plate. Furthermore, the subacute toxicity assessment was conducted in accordance with OECD guidelines, wherein the rats received the extract at the doses of 70, 350, and 1000 mg/kg/day. Although there were some significant variations in some evaluated parameters, they were not specifically linked to the extract. As no treatment‐related and toxicologically significant adverse effects were noticed up to the dose of 1000 mg/kg/day, the NOAEL for the extract was determined to be 1000 mg/kg/day. Renogrit, however, contains the extracts of the whole plant of *Cichorium intybus* L., which includes the roots as well.

Furthermore, an aqueous extract prepared from the fruits of *Tribulus terrestris* L. has also been tested for its nonclinical safety profile subsequent to repeated oral administration for 28 consecutive days, at the doses of 75, 225, and 750 mg/kg/day [40]. This OECD‐driven study concluded that despite some statistically significant changes in certain study parameters, the extract was judged to be safe up to the highest tested dose, as the observed variations were non–dose‐dependent and incidental. Accordingly, its NOAEL was considered to be 750 mg/kg/day. In this study, even the maximum dose, which the rats would have received for 28 successive days, is again quite low (132.4 mg/kg/day). Taken together, the available literature references covering safety evaluations of the extracts of botanical drugs present in Renogrit suggest that the maximum doses received by the rats in our study are only a fraction of the NOAELs reported for these phytoconstituents and accordingly support the safety profile of Renogrit.

An identified limitation of the current nonclinical safety study is the absence of toxicokinetic data. From the perspective of small molecule‐based drugs, reporting of their toxicokinetic parameters utilizing satellite groups is imperative. However, in case of botanical drug–based medicines, which often contain several phytoconstituents, such a determination is an arduous task. This difficulty fundamentally arises due to the presence of structurally complex and diverse phytometabolites, which are present in slight amounts. Furthermore, this study has only reported the mutagenic potential of Renogrit in bacteria and its 28‐day repeated dose safety in rodents. Forthcoming studies are necessary which should comprehensively evaluate the complete in vitro and in vivo genetic safety of Renogrit as per the established OECD guidance in mammalian systems. In addition, in vivo nonclinical safety assessments of Renogrit should also encompass subchronic (90‐day repeated oral administration) and chronic (180‐day repeated oral administration) toxicity tests in rodents. Likewise, reproductive toxicity assessments of Renogrit in rodents are also warranted. Moreover, future experiments should also be planned which determine the in vivo safety of Renogrit in larger animals. These regulatory nonclinical safety evaluations would ultimately provide the robust foundation for the detailed evaluation of Renogrit in humans in clinical settings.

## 5. Conclusion

The mutagenicity of Renogrit was tested with the Ames assay using histidine‐dependent *S. typhimurium* tester strains, TA 98, TA 100, TA 1537, and TA 1535, and tryptophan‐dependent *E. coli uvrA* in the presence and absence of metabolic activation with rat liver S9 fraction as per the OECD Test Guideline 471. Renogrit was tested up to 5.0 mg/plate; however, it did not demonstrate any mutagenic effect in any of the *S. typhimurium* and *E. coli uvrA* strains at all the tested concentrations. The nonclinical subacute toxicity evaluation of Renogrit was conducted in accordance with the OECD Test Guideline 407, involving repeated oral administration to male and female SD rats at doses of 100, 300, and 1000 mg/kg/day over a 28‐day period. The study design also included a recovery cohort, wherein animals receiving the vehicle or the highest dose of Renogrit were monitored for an additional 14 days following cessation of treatment to assess the potential for delayed, persistent, or reversible toxic effects. Across all evaluated parameters, including clinical observations, body weight, feed consumption, ophthalmological examination, hematology, clinical biochemistry, organ weights, gross pathology observations, and histopathology, Renogrit did not elicit any adverse or detrimental systemic effect which could be attributed to the Ayurvedic medicine.

Based on these findings, Renogrit is found to be nonmutagenic, and the NOAEL for Renogrit in rats was established to be 1000 mg/kg/day in both male and female rats. The study outcomes correspondingly back its future safety investigations in rodents for a longer duration and in larger animals as well. Additionally, the conclusions also serve as a first step toward its detailed clinical evaluation.

## Author Contributions

Acharya Balkrishna: conceptualization, supervision, visualization, and writing–review and editing. Aakanksha Tiwari: conceptualization, planning, visualization, methodology, investigation, data curation, and formal analysis. Himanshu Jangid: methodology, investigation, and formal analysis. Kamaraj Mani: data curation, formal analysis, and writing–original draft. Savita Lochab: data curation, visualization, project administration, supervision, and writing–review and editing. Sandeep Sinha: data curation, visualization, project administration, supervision, and writing–review and editing. Anurag Varshney: conceptualization, project administration, supervision, visualization, and writing–review and editing.

## Funding

No external funding was received for this study. This research work was supported by internal funds from Patanjali Research Foundation Trust, Haridwar, India.

## Ethics Statement

The testing facility, Patanjali Research Foundation, Haridwar, is certified by the CCSEA (Registration Number: 1964/PO/Rc/S/17/CPCSEA), Department of Animal Husbandry and Dairying, Ministry of Fisheries, Animal Husbandry and Dairying, Government of India, for conducting experiments on small laboratory animals. The experimental protocol (Proposal No. PRIAS/LAF/IAEC‐149) was meticulously reviewed and received approval from the IAEC of Patanjali Research Foundation.

## Conflicts of Interest

Acharya Balkrishna is an honorary trustee in the Divya Yog Mandir Trust, which governs Divya Pharmacy, Haridwar, Uttarakhand, India. The test formulation, Renogrit, was sourced from Divya Pharmacy. Renogrit is a marketed medicinal product of Divya Pharmacy. In addition, Acharya Balkrishna holds an honorary managerial position in Patanjali Ayurved Ltd., Haridwar, India. Divya Pharmacy and Patanjali Ayurved Ltd. manufacture and sell many herbal medicinal products. Other than providing the test formulation Renogrit, Divya Pharmacy and Patanjali Ayurved Ltd. were not involved in any aspect of the research reported in this study. The other authors declare no conflicts of interest.

## Supporting Information

SUPPORTING TABLE 1: Effect of Renogrit on weekly body weight gain of rats w.r.t Day 1. Data presented as Mean ± Standard Deviation (*n* = 5) and was statistically analysed by employing two‐way ANOVA followed by Tukey’s multiple comparison test. G1 (0); Vehicle control animals received 0.5% Methyl cellulose (10 mL/kg/day), G2 (100), G3 (300), G4 (1000): Experimental animals received Renogrit at corresponding doses of 100, 300 and 1000 mg/kg/day for 28 consecutive days. G1R (0): Vehicle recovery group animals, observed 14 days after withdrawing 28 days 0.5% Methyl cellulose treatment. G4R (1000): Renogrit recovery group animals, observed 14 days after withdrawing 28 days Renogrit treatment.

SUPPORTING TABLE 2: General clinical signs, morbidity and mortality data. Data presented as number of animal observed for morbidity/mortality and total number of animals. (*n* = Number of animal). G1 (0); Vehicle control animals received 0.5% Methyl cellulose (10 mL/kg/day), G2 (100), G3 (300), G4 (1000): Experimental animals received Renogrit at corresponding doses of 100, 300 and 1000 mg/kg/day for 28 consecutive days. G1R (0): Vehicle recovery group animals, observed 14 days after withdrawing 28 days 0.5% Methyl cellulose treatment. G4R (1000): Renogrit recovery group animals, observed 14 days after withdrawing 28 days Renogrit treatment.

SUPPORTING TABLE 3: Detailed clinical observations following administration of vehicle/Renogrit. Data presented as number of animal showed no clinical observation and total number of animals. G1 (0); Vehicle control animals received 0.5% Methyl cellulose (10 mL/kg/day), G2 (100), G3 (300), G4 (1000): Experimental animals received Renogrit at corresponding doses of 100, 300 and 1000 mg/kg/day for 28 consecutive days. G1R (0): Vehicle recovery group animals, observed 14 days after withdrawing 28 days 0.5% Methyl cellulose treatment. G4R (1000): Renogrit recovery group animals, observed 14 days after withdrawing 28 days Renogrit treatment.

SUPPORTING TABLE 4: Functional observation battery (behavioral responses) in rats that were administered with vehicle/Renogrit. Data presented as number of animal showed no behavioral responses and total number of animals. G1 (0); Vehicle control animals received 0.5% Methyl cellulose (10 mL/kg/day), G2 (100), G3 (300), G4 (1000): Experimental animals received Renogrit at corresponding doses of 100, 300 and 1000 mg/kg/day for 28 consecutive days. G1R (0): Vehicle recovery group animals, observed 14 days after withdrawing 28 days 0.5% Methyl cellulose treatment. G4R (1000): Renogrit recovery group animals, observed 14 days after withdrawing 28 days Renogrit treatment.

SUPPORTING TABLE 5: Functional observation battery (neurologic responses) in rats that were administered with vehicle/Renogrit. Data presented as number of animal showed no neurologic responses and total number of animals. G1 (0); Vehicle control animals received 0.5% Methyl cellulose (10 mL/kg/day), G2 (100), G3 (300), G4 (1000): Experimental animals received Renogrit at corresponding doses of 100, 300 and 1000 mg/kg/day for 28 consecutive days. G1R (0): Vehicle recovery group animals, observed 14 days after withdrawing 28 days 0.5% Methyl cellulose treatment. G4R (1000): Renogrit recovery group animals, observed 14 days after withdrawing 28 days Renogrit treatment.

SUPPORTING TABLE 6: Functional observation battery (autonomic responses) in rats that were administered with vehicle/Renogrit. Data presented as number of animal showed no autonomic responses and total number of animals. G1 (0); Vehicle control animals received 0.5% Methyl cellulose (10 mL/kg/day), G2 (100), G3 (300), G4 (1000): Experimental animals received Renogrit at corresponding doses of 100, 300 and 1000 mg/kg/day for 28 consecutive days. G1R (0): Vehicle recovery group animals, observed 14 days after withdrawing 28 days 0.5% Methyl cellulose treatment. G4R (1000): Renogrit recovery group animals, observed 14 days after withdrawing 28 days Renogrit treatment.

SUPPORTING TABLE 7: Ophthalmoscopic examination of male and female rats that received vehicle/Renogrit. Data presented as number of animal showed NAD and total number of animals. G1 (0); Vehicle control animals received 0.5% Methyl cellulose (10 mL/kg/day), G2 (100), G3 (300), G4 (1000): Experimental animals received Renogrit at corresponding doses of 100, 300 and 1000 mg/kg/day for 28 consecutive days. G1R (0): Vehicle recovery group animals, observed 14 days after withdrawing 28 days 0.5% Methyl cellulose treatment. G4R (1000): Renogrit recovery group animals, observed 14 days after withdrawing 28 days Renogrit treatment.

SUPPORTING TABLE 8: Effect of Renogrit on absolute organ weights (in g). Data presented as Mean ± Standard Deviation (*n* = 5) and was statistically analysed by employing two‐way ANOVA followed by Dunnett’s multiple comparison test. G1 (0); Vehicle control animals received 0.5% Methyl cellulose (10 mL/kg/day), G2 (100), G3 (300), G4 (1000): Experimental animals received Renogrit at corresponding doses of 100, 300 and 1000 mg/kg/day for 28 consecutive days. G1R (0): Vehicle recovery group animals, observed 14 days after withdrawing 28 days 0.5% Methyl cellulose treatment. G4R (1000): Renogrit recovery group animals, observed 14 days after withdrawing 28 days Renogrit treatment.

SUPPORTING TABLE 9: Effect of Renogrit on relative organ weights represented as percentage of terminal brain weights. Data presented as Mean ± Standard Deviation (*n* = 5) and was statistically analysed by employing two‐way ANOVA followed by Dunnett’s multiple comparison test. G1 (0); Vehicle control animals received 0.5% Methyl cellulose (10 mL/kg/day), G2 (100), G3 (300), G4 (1000): Experimental animals received Renogrit at corresponding doses of 100, 300 and 1000 mg/kg/day for 28 consecutive days. G1R (0): Vehicle recovery group animals, observed 14 days after withdrawing 28 days 0.5% Methyl cellulose treatment. G4R (1000): Renogrit recovery group animals, observed 14 days after withdrawing 28 days Renogrit treatment.

## Supporting information


**Supporting Information** Additional supporting information can be found online in the Supporting Information section.

## Data Availability

The study data will be provided upon reasonable request from the corresponding author.
