# Supplementary material for: Nonclinical Safety Evaluation of Renogrit: In Vitro Nonmutagenicity (OECD 471) and 28‐Day Repeated Oral Dose Toxicity (OECD 407) in Sprague Dawley Rats
Source: J Toxicol. 2026 Apr 24;2026:5905101. doi: 10.1155/jt/5905101 (PMC13107116; doi:10.1155/jt/5905101)
Supplement: Supplementary file 1 — Supporting Information Additional supporting information can be found online in the Supporting Information section. [file JT-2026-5905101-s001.docx]

**SUPPLEMENTARY MATERIAL**

This supplementary material contains following tables:

TABLE 1: Effect of Renogrit on weekly body weight gain of rats w.r.t Day 1

TABLE 2: General clinical signs, morbidity and mortality data

TABLE 3: Detailed clinical observations following administration of vehicle/Renogrit

TABLE 4: Functional observation battery (behavioral responses) in rats that were administered with vehicle/Renogrit

TABLE 5: Functional observation battery (neurologic responses) in rats that were administered with vehicle/Renogrit

TABLE 6: Functional observation battery (autonomic responses) in rats that were administered with vehicle/Renogrit

TABLE 7: Ophthalmoscopic examination of male and female rats that received vehicle/Renogrit

TABLE 8: Effect of Renogrit on absolute organ weights (in g)

TABLE 9: Effect of Renogrit on relative organ weights represented as percentage of terminal brain weights.

SUPPL. TABLE 1: Effect of Renogrit on weekly body weight gain of rats w.r.t Day 1

| **Treatment** | **28 days treatment (mg/kg/day)** | | | | **14 days recovery (mg/kg/day)** | |
| --- | --- | --- | --- | --- | --- | --- |
|  | **G1 (0)** | **G2 (100)** | **G3 (300)** | **G4 (1000)** | **G1R (0)** | **G4R (1000)** |
| **Male (n=5)** | | | | | | |
| **Day 8** | 54.56±7.80 | 55.43±5.76 | 58.80±4.55 | 59.51±10.16 | 56.97±6.75 | 53.42±5.36 |
| **Day 15** | 104.38±18.30 | 100.25±10.52 | 114.45±13.70 | 104.27±15.88 | 102.29±20.33 | 97.62±11.24 |
| **Day 22** | 137.03±33.65 | 130.96±7.69 | 152.52±19.70 | 147.20±22.22 | 139.17±24.32 | 129.02±26.39 |
| **Day 28** | 168.70±37.87 | 159.12±9.92 | 186.32±20.28 | 178.03±27.72 | 169.90±27.39 | 155.36±34.72 |
| **Day 35** | NA | NA | NA | NA | 195.49±32.64 | 184.56±33.73 |
| **Day 42** | NA | NA | NA | NA | 209.44±39.99 | 208.61±39.09 |
| **Female (n=5)** | | | | | | |
| **Day 8** | 16.19±7.62 | 14.30±8.67 | 16.90±10.41 | 11.73±11.61 | 17.36±11.43 | 18.62±7.41 |
| **Day 15** | 38.12±8.48 | 35.31±5.09 | 47.09±8.24 | 33.93±7.66 | 36.81±16.39 | 37.25±4.01 |
| **Day 22** | 53.65±6.36 | 52.70±6.91 | 63.52±10.99 | 48.84±13.65 | 51.83±13.99 | 56.81±10.15 |
| **Day 28** | 62.68±10.84 | 64.61±5.06 | 71.25±15.93 | 58.20±18.61 | 69.67±20.93 | 65.13±7.92 |
| **Day 35** | NA | NA | NA | NA | 71.69±22.47 | 71.53±7.63 |
| **Day 42** | NA | NA | NA | NA | 74.25±20.83 | 76.38±12.49 |

*Note*: Data presented as Mean ± Standard Deviation (n = 5) and was statistically analysed by employing two-way ANOVA followed by Tukey’s multiple comparison test. G1 (0); Vehicle control animals received 0.5% Methyl cellulose (10mL/kg/day), G2 (100), G3 (300), G4 (1000): Experimental animals received Renogrit at corresponding doses of 100, 300 and 1000 mg/kg/day for 28 consecutive days. G1R (0): Vehicle recovery group animals, observed 14 days after withdrawing 28 days 0.5% Methyl cellulose treatment. G4R (1000): Renogrit recovery group animals, observed 14 days after withdrawing 28 days Renogrit treatment.

No significant changes in body weight gain were noticed between the vehicle and Renogrit treated groups both in the main and recovery arms in male and female animals

SUPPL. TABLE 2: General clinical signs, morbidity and mortality data

| **Parameter** | **28 days treatment (mg/kg/day)** | | | | **14 days recovery (mg/kg/day)** | |
| --- | --- | --- | --- | --- | --- | --- |
|  | **G1 (0)** | **G2 (100)** | **G3 (300)** | **G4 (1000)** | **G1R (0)** | **G4R (1000)** |
| **Male (n=5)** | | | | | | |
| **General Clinical Signs** | Absent | Absent | Absent | Absent | Absent | Absent |
| **Morbidity** | 0/5 | 0/5 | 0/5 | 0/5 | 0/5 | 0/5 |
| **Mortality** | 0/5 | 0/5 | 0/5 | 0/5 | 0/5 | 0/5 |
| **Female (n=5)** | | | | | | |
| **General Clinical Signs** | Absent | Absent | Absent | Absent | Absent | Absent |
| **Morbidity** | 0/5 | 0/5 | 0/5 | 0/5 | 0/5 | 0/5 |
| **Mortality** | 0/5 | 0/5 | 0/5 | 0/5 | 0/5 | 0/5 |

*Note*: Data presented as number of animal observed for morbidity/mortality and total number of animals. (n=Number of animal). G1 (0); Vehicle control animals received 0.5% Methyl cellulose (10mL/kg/day), G2 (100), G3 (300), G4 (1000): Experimental animals received Renogrit at corresponding doses of 100, 300 and 1000 mg/kg/day for 28 consecutive days. G1R (0): Vehicle recovery group animals, observed 14 days after withdrawing 28 days 0.5% Methyl cellulose treatment. G4R (1000): Renogrit recovery group animals, observed 14 days after withdrawing 28 days Renogrit treatment.

No abnormal clinical signs, morbidity, or mortality were observed in any of the groups (control and Renogrit-treated groups at 100, 300, and 1000 mg/kg/day) in either male or female animals

SUPPL. TABLE 3: Detailed clinical observations following administration of vehicle/Renogrit

| **Treatment** | **28 days treatment (mg/kg/day)** | | | | **14 days recovery (mg/kg/day)** | |
| --- | --- | --- | --- | --- | --- | --- |
|  | **G1 (0)** | **G2 (100)** | **G3 (300)** | **G4 (1000)** | **G1R (0)** | **G4R (1000)** |
| **Male (n=5)** | | | | | | |
| **Day 1** | NAD (5/5) | NAD (5/5) | NAD (5/5) | NAD (5/5) | NAD (5/5) | NAD (5/5) |
| **Day 8** | NAD (5/5) | NAD (5/5) | NAD (5/5) | NAD (5/5) | NAD (5/5) | NAD (5/5) |
| **Day 15** | NAD (5/5) | NAD (5/5) | NAD (5/5) | NAD (5/5) | NAD (5/5) | NAD (5/5) |
| **Day 22** | NAD (5/5) | NAD (5/5) | NAD (5/5) | NAD (5/5) | NAD (5/5) | NAD (5/5) |
| **Day 28** | NAD (5/5) | NAD (5/5) | NAD (5/5) | NAD (5/5) | NAD (5/5) | NAD (5/5) |
| **Day 35** | NA | NA | NA | NA | NAD (5/5) | NAD (5/5) |
| **Day 42** | NA | NA | NA | NA | NAD (5/5) | NAD (5/5) |
| **Female (n=5)** | | | | | | |
| **Day 1** | NAD (5/5) | NAD (5/5) | NAD (5/5) | NAD (5/5) | NAD (5/5) | NAD (5/5) |
| **Day 8** | NAD (5/5) | NAD (5/5) | NAD (5/5) | NAD (5/5) | NAD (5/5) | NAD (5/5) |
| **Day 15** | NAD (5/5) | NAD (5/5) | NAD (5/5) | NAD (5/5) | NAD (5/5) | NAD (5/5) |
| **Day 22** | NAD (5/5) | NAD (5/5) | NAD (5/5) | NAD (5/5) | NAD (5/5) | NAD (5/5) |
| **Day 28** | NAD (5/5) | NAD (5/5) | NAD (5/5) | NAD (5/5) | NAD (5/5) | NAD (5/5) |
| **Day 35** | NA | NA | NA | NA | NAD (5/5) | NAD (5/5) |
| **Day 42** | NA | NA | NA | NA | NAD (5/5) | NAD (5/5) |

NAD = No Abnormality Detected; NA: Not Applicable; n=Number of animals

*Note*: Data presented as number of animal showed no clinical observation and total number of animals. G1 (0); Vehicle control animals received 0.5% Methyl cellulose (10mL/kg/day), G2 (100), G3 (300), G4 (1000): Experimental animals received Renogrit at corresponding doses of 100, 300 and 1000 mg/kg/day for 28 consecutive days. G1R (0): Vehicle recovery group animals, observed 14 days after withdrawing 28 days 0.5% Methyl cellulose treatment. G4R (1000): Renogrit recovery group animals, observed 14 days after withdrawing 28 days Renogrit treatment.

All experimental animals appeared clinically normal during the detailed weekly examinations across the entire study duration, indicating no overt signs of toxicity associated with Renogrit administration.

SUPPL. TABLE 4: Functional observation battery (behavioral responses) in rats that were administered with vehicle/Renogrit

| **Parameter** | | **28 days treatment (mg/kg/day)** | | | | | | **14 days recovery (mg/kg/day)** | |
| --- | --- | --- | --- | --- | --- | --- | --- | --- | --- |
|  |  | **G1 (0)** | **G2 (100)** | **G3 (300)** | **G4 (1000)** | **G1R (0)** | **G4R (1000)** | **G1 (0)** | **G2 (100)** |
| **Males (n=5)** | | | | | | | | | |
| **Behavioral Responses** | Posture | NAD (5/5) | NAD (5/5) | NAD (5/5) | NAD (5/5) | NAD (5/5) | NAD (5/5) | NAD (5/5) | NAD (5/5) |
|  | Ease of removal | NAD (5/5) | NAD (5/5) | NAD (5/5) | NAD (5/5) | NAD (5/5) | NAD (5/5) | NAD (5/5) | NAD (5/5) |
|  | Ease of handling | NAD (5/5) | NAD (5/5) | NAD (5/5) | NAD (5/5) | NAD (5/5) | NAD (5/5) | NAD (5/5) | NAD (5/5) |
|  | Alertness | NAD (5/5) | NAD (5/5) | NAD (5/5) | NAD (5/5) | NAD (5/5) | NAD (5/5) | NAD (5/5) | NAD (5/5) |
|  | Mobility | NAD (5/5) | NAD (5/5) | NAD (5/5) | NAD (5/5) | NAD (5/5) | NAD (5/5) | NAD (5/5) | NAD (5/5) |
|  | Fur appearance | NAD (5/5) | NAD (5/5) | NAD (5/5) | NAD (5/5) | NAD (5/5) | NAD (5/5) | NAD (5/5) | NAD (5/5) |
|  | Approach response | NAD (5/5) | NAD (5/5) | NAD (5/5) | NAD (5/5) | NAD (5/5) | NAD (5/5) | NAD (5/5) | NAD (5/5) |
|  | Touch response | NAD (5/5) | NAD (5/5) | NAD (5/5) | NAD (5/5) | NAD (5/5) | NAD (5/5) | NAD (5/5) | NAD (5/5) |
|  | Startle response | NAD (5/5) | NAD (5/5) | NAD (5/5) | NAD (5/5) | NAD (5/5) | NAD (5/5) | NAD (5/5) | NAD (5/5) |
| **Females (n=5)** | | | | | | | | | |
| **Behavioral Responses** | Posture | NAD (5/5) | NAD (5/5) | NAD (5/5) | NAD (5/5) | NAD (5/5) | NAD (5/5) | NAD (5/5) | NAD (5/5) |
|  | Ease of removal | NAD (5/5) | NAD (5/5) | NAD (5/5) | NAD (5/5) | NAD (5/5) | NAD (5/5) | NAD (5/5) | NAD (5/5) |
|  | Ease of handling | NAD (5/5) | NAD (5/5) | NAD (5/5) | NAD (5/5) | NAD (5/5) | NAD (5/5) | NAD (5/5) | NAD (5/5) |
|  | Alertness | NAD (5/5) | NAD (5/5) | NAD (5/5) | NAD (5/5) | NAD (5/5) | NAD (5/5) | NAD (5/5) | NAD (5/5) |
|  | Mobility | NAD (5/5) | NAD (5/5) | NAD (5/5) | NAD (5/5) | NAD (5/5) | NAD (5/5) | NAD (5/5) | NAD (5/5) |
|  | Fur appearance | NAD (5/5) | NAD (5/5) | NAD (5/5) | NAD (5/5) | NAD (5/5) | NAD (5/5) | NAD (5/5) | NAD (5/5) |
|  | Approach response | NAD (5/5) | NAD (5/5) | NAD (5/5) | NAD (5/5) | NAD (5/5) | NAD (5/5) | NAD (5/5) | NAD (5/5) |
|  | Touch response | NAD (5/5) | NAD (5/5) | NAD (5/5) | NAD (5/5) | NAD (5/5) | NAD (5/5) | NAD (5/5) | NAD (5/5) |
|  | Startle response | NAD (5/5) | NAD (5/5) | NAD (5/5) | NAD (5/5) | NAD (5/5) | NAD (5/5) | NAD (5/5) | NAD (5/5) |

N= Number of animals; NAD *=* No Abnormality Detected.

*Note*: Data presented as number of animal showed no behavioral responses and total number of animals. G1 (0); Vehicle control animals received 0.5% Methyl cellulose (10mL/kg/day), G2 (100), G3 (300), G4 (1000): Experimental animals received Renogrit at corresponding doses of 100, 300 and 1000 mg/kg/day for 28 consecutive days. G1R (0): Vehicle recovery group animals, observed 14 days after withdrawing 28 days 0.5% Methyl cellulose treatment. G4R (1000): Renogrit recovery group animals, observed 14 days after withdrawing 28 days Renogrit treatment.

Behavioral responses assessed through the functional observational battery, were normal across all male and female subjects in both study phases

SUPPL. TABLE 5: Functional observation battery (neurologic responses) in rats that were administered with vehicle/Renogrit

| **Parameter** | | **28 days treatment (mg/kg/day)** | | | | | | **14 days recovery (mg/kg/day)** | |
| --- | --- | --- | --- | --- | --- | --- | --- | --- | --- |
|  |  | **G1 (0)** | **G2 (100)** | **G3 (300)** | **G4 (1000)** | **G1R (0)** | **G4R (1000)** | **G1 (0)** | **G2 (100)** |
| **Males (n=5)** | | | | | | | | | |
| **Neurologic Responses** | Convulsions and tremors | Absent (5/5) | Absent (5/5) | Absent (5/5) | Absent (5/5) | Absent (5/5) | Absent (5/5) | Absent (5/5) | Absent (5/5) |
|  | Muscle tone | NAD (5/5) | NAD (5/5) | NAD (5/5) | NAD (5/5) | NAD (5/5) | NAD (5/5) | NAD (5/5) | NAD (5/5) |
|  | Gait | NAD (5/5) | NAD (5/5) | NAD (5/5) | NAD (5/5) | NAD (5/5) | NAD (5/5) | NAD (5/5) | NAD (5/5) |
|  | Stereotypic movements | Absent (5/5) | Absent (5/5) | Absent (5/5) | Absent (5/5) | Absent (5/5) | Absent (5/5) | Absent (5/5) | Absent (5/5) |
|  | Tail pinch response | NAD (5/5) | NAD (5/5) | NAD (5/5) | NAD (5/5) | NAD (5/5) | NAD (5/5) | NAD (5/5) | NAD (5/5) |
|  | Eye blink response | NAD (5/5) | NAD (5/5) | NAD (5/5) | NAD (5/5) | NAD (5/5) | NAD (5/5) | NAD (5/5) | NAD (5/5) |
|  | Air righting reflex | NAD (5/5) | NAD (5/5) | NAD (5/5) | NAD (5/5) | NAD (5/5) | NAD (5/5) | NAD (5/5) | NAD (5/5) |
| **Females (n=5)** | | | | | | | | | |
| **Neurologic Responses** | Convulsions and tremors | Absent (5/5) | Absent (5/5) | Absent (5/5) | Absent (5/5) | Absent (5/5) | Absent (5/5) | Absent (5/5) | Absent (5/5) |
|  | Muscle tone | NAD (5/5) | NAD (5/5) | NAD (5/5) | NAD (5/5) | NAD (5/5) | NAD (5/5) | NAD (5/5) | NAD (5/5) |
|  | Gait | NAD (5/5) | NAD (5/5) | NAD (5/5) | NAD (5/5) | NAD (5/5) | NAD (5/5) | NAD (5/5) | NAD (5/5) |
|  | Stereotypic movements | Absent (5/5) | Absent (5/5) | Absent (5/5) | Absent (5/5) | Absent (5/5) | Absent (5/5) | Absent (5/5) | Absent (5/5) |
|  | Tail pinch response | NAD (5/5) | NAD (5/5) | NAD (5/5) | NAD (5/5) | NAD (5/5) | NAD (5/5) | NAD (5/5) | NAD (5/5) |
|  | Eye blink response | NAD (5/5) | NAD (5/5) | NAD (5/5) | NAD (5/5) | NAD (5/5) | NAD (5/5) | NAD (5/5) | NAD (5/5) |
|  | Air righting reflex | NAD (5/5) | NAD (5/5) | NAD (5/5) | NAD (5/5) | NAD (5/5) | NAD (5/5) | NAD (5/5) | NAD (5/5) |

n = Number of animals; NAD *=* No Abnormality Detected

*Note*: Data presented as number of animal showed no neurologic responses and total number of animals. G1 (0); Vehicle control animals received 0.5% Methyl cellulose (10mL/kg/day), G2 (100), G3 (300), G4 (1000): Experimental animals received Renogrit at corresponding doses of 100, 300 and 1000 mg/kg/day for 28 consecutive days. G1R (0): Vehicle recovery group animals, observed 14 days after withdrawing 28 days 0.5% Methyl cellulose treatment. G4R (1000): Renogrit recovery group animals, observed 14 days after withdrawing 28 days Renogrit treatment.

Neurological examinations revealed no abnormalities in muscle tone, gait, tail pinch response, olfactory response, eye blink response, or air righting reflex in either the main or recovery groups

SUPPL. TABLE 6: Functional observation battery (autonomic responses) in rats that were administered with vehicle/Renogrit

| **Parameter** | | **28 days treatment (mg/kg/day)** | | | | | | **14 days recovery (mg/kg/day)** | | |
| --- | --- | --- | --- | --- | --- | --- | --- | --- | --- | --- |
|  |  | **G1 (0)** | **G2 (100)** | **G3 (300)** | **G4 (1000)** | **G1R (0)** | **G4R (1000)** | **G1 (0)** | **G2 (100)** |  |
| **Males (n=5)** | | | | | | | | | | |
| **Autonomic response** | Palpebral closure | NAD (5/5) | NAD (5/5) | NAD (5/5) | NAD (5/5) | NAD (5/5) | NAD (5/5) | NAD (5/5) | NAD (5/5) |  |
|  | Lacrimation | NAD (5/5) | NAD (5/5) | NAD (5/5) | NAD (5/5) | NAD (5/5) | NAD (5/5) | NAD (5/5) | NAD (5/5) |  |
|  | Salivation | NAD (5/5) | NAD (5/5) | NAD (5/5) | NAD (5/5) | NAD (5/5) | NAD (5/5) | NAD (5/5) | NAD (5/5) |  |
|  | Eye colour | NAD (5/5) | NAD (5/5) | NAD (5/5) | NAD (5/5) | NAD (5/5) | NAD (5/5) | NAD (5/5) | NAD (5/5) |  |
|  | Pupil function in response to light | NAD (5/5) | NAD (5/5) | NAD (5/5) | NAD (5/5) | NAD (5/5) | NAD (5/5) | NAD (5/5) | NAD (5/5) |  |
|  | Skin colour | NAD (5/5) | NAD (5/5) | NAD (5/5) | NAD (5/5) | NAD (5/5) | NAD (5/5) | NAD (5/5) | NAD (5/5) |  |
|  | Piloerection | Absent (5/5) | Absent (5/5) | Absent (5/5) | Absent (5/5) | Absent (5/5) | Absent (5/5) | Absent (5/5) | Absent (5/5) |  |
|  | Ease and rate of respiration | NAD (5/5) | NAD (5/5) | NAD (5/5) | NAD (5/5) | NAD (5/5) | NAD (5/5) | NAD (5/5) | NAD (5/5) |  |
|  | Faecal consistency | NAD (5/5) | NAD (5/5) | NAD (5/5) | NAD (5/5) | NAD (5/5) | NAD (5/5) | NAD (5/5) | NAD (5/5) |  |
| **Females (n=5)** | | | | | | | | | | |
| **Autonomic response** | Palpebral closure | NAD (5/5) | NAD (5/5) | NAD (5/5) | NAD (5/5) | NAD (5/5) | NAD (5/5) | NAD (5/5) | NAD (5/5) |  |
|  | Lacrimation | NAD (5/5) | NAD (5/5) | NAD (5/5) | NAD (5/5) | NAD (5/5) | NAD (5/5) | NAD (5/5) | NAD (5/5) |  |
|  | Salivation | NAD (5/5) | NAD (5/5) | NAD (5/5) | NAD (5/5) | NAD (5/5) | NAD (5/5) | NAD (5/5) | NAD (5/5) |  |
|  | Eye colour | NAD (5/5) | NAD (5/5) | NAD (5/5) | NAD (5/5) | NAD (5/5) | NAD (5/5) | NAD (5/5) | NAD (5/5) |  |
|  | Pupil function in response to light | NAD (5/5) | NAD (5/5) | NAD (5/5) | NAD (5/5) | NAD (5/5) | NAD (5/5) | NAD (5/5) | NAD (5/5) |  |
|  | Skin colour | NAD (5/5) | NAD (5/5) | NAD (5/5) | NAD (5/5) | NAD (5/5) | NAD (5/5) | NAD (5/5) | NAD (5/5) |  |
|  | Piloerection | Absent (5/5) | Absent (5/5) | Absent (5/5) | Absent (5/5) | Absent (5/5) | Absent (5/5) | Absent (5/5) | Absent (5/5) |  |
|  | Ease and rate of respiration | NAD (5/5) | NAD (5/5) | NAD (5/5) | NAD (5/5) | NAD (5/5) | NAD (5/5) | NAD (5/5) | NAD (5/5) |  |
|  | Faecal consistency | NAD (5/5) | NAD (5/5) | NAD (5/5) | NAD (5/5) | NAD (5/5) | NAD (5/5) | NAD (5/5) | NAD (5/5) |  |

n= Number of animals; NAD *=* No Abnormality Detected

*Note*: Data presented as number of animal showed no autonomic responses and total number of animals. G1 (0); Vehicle control animals received 0.5% Methyl cellulose (10mL/kg/day), G2 (100), G3 (300), G4 (1000): Experimental animals received Renogrit at corresponding doses of 100, 300 and 1000 mg/kg/day for 28 consecutive days. G1R (0): Vehicle recovery group animals, observed 14 days after withdrawing 28 days 0.5% Methyl cellulose treatment. G4R (1000): Renogrit recovery group animals, observed 14 days after withdrawing 28 days Renogrit treatment.

Autonomic responses were within normal limits in all animals throughout the functional observational battery assessments, with no abnormalities detected in either main or recovery groups

SUPPL. TABLE 7: Ophthalmoscopic examination of male and female rats that received vehicle/Renogrit

| **Eye examined** | **28 day Renogrit treatment (mg/kg/day)** | | | | | | **14-day recovery (mg/kg/day)** | |
| --- | --- | --- | --- | --- | --- | --- | --- | --- |
|  | **Week 4** | | | | | | **Week 6** | |
|  | **G1 (0)** | **G2 (100)** | **G3 (300)** | **G4 (1000)** | **G1R (0)** | **G4R (1000)** | **G1R (0)** | **G4R (1000)** |
| **Males (n=5)** | | | | | | | | |
| **Left Eye** | NAD (5/5) | NAD (5/5) | NAD (5/5) | NAD (5/5) | NAD (5/5) | NAD (5/5) | NAD (5/5) | NAD (5/5) |
| **Right Eye** | NAD (5/5) | NAD (5/5) | NAD (5/5) | NAD (5/5) | NAD (5/5) | NAD (5/5) | NAD (5/5) | NAD (5/5) |
| **Females (n=5)** | | | | | | | | |
| **Left Eye** | NAD (5/5) | NAD (5/5) | NAD (5/5) | NAD (5/5) | NAD (5/5) | NAD (5/5) | NAD (5/5) | NAD (5/5) |
| **Right Eye** | NAD (5/5) | NAD (5/5) | NAD (5/5) | NAD (5/5) | NAD (5/5) | NAD (5/5) | NAD (5/5) | NAD (5/5) |

N = Number of animals; NAD *=* No Abnormality Detected

*Note*: Data presented as number of animal showed NAD and total number of animals. G1 (0); Vehicle control animals received 0.5% Methyl cellulose (10mL/kg/day), G2 (100), G3 (300), G4 (1000): Experimental animals received Renogrit at corresponding doses of 100, 300 and 1000 mg/kg/day for 28 consecutive days. G1R (0): Vehicle recovery group animals, observed 14 days after withdrawing 28 days 0.5% Methyl cellulose treatment. G4R (1000): Renogrit recovery group animals, observed 14 days after withdrawing 28 days Renogrit treatment.

Renogrit administration for 28-consecutive days did not lead to any ocular abnormality in both male and female animals, when examined during week 4, in both main and recovery arms. During week 6, ophthalmoscopic examination of the eyes of both male and female rats, allocated to the recovery arms, did not reveal any delayed onset of aberrant ocular findings.

SUPPL. TABLE 8: Effect of Renogrit on absolute organ weights (in g)

| **Parameter** | **28 days treatment (mg/kg/day)** | | | | **14 days recovery (mg/kg/day)** | |
| --- | --- | --- | --- | --- | --- | --- |
|  | **G1 (0)** | **G2 (100)** | **G3 (300)** | **G4 (1000)** | **G1R (0)** | **G4R (1000)** |
| **Male (n=5)** | | | | | | |
| Liver | 16.31±2.335 | 15.24±2.257 | 18.57±2.424 | 17.12±2.573 | 20.21±2.769 | 17.90±2.039 |
| Kidneys | 3.19±0.254 | 2.99±0.251 | 3.41±0.219 | 3.35±0.317 | 3.79±0.496 | 3.63±0.611 |
| Adrenals | 0.07±0.008 | 0.06±0.021 | 0.06±0.011 | 0.07±0.017 | 0.08±0.019 | 0.07±0.015 |
| Spleen | 0.80±0.125 | 0.78±0.149 | 0.83±0.095 | 0.81±0.133 | 0.79±0.126 | 0.87±0.168 |
| Brain | 2.16±0.074 | 2.07±0.107 | 2.05±0.091 | 2.03±0.062 | 2.11±0.149 | 2.15±0.101 |
| Heart | 1.40±0.111 | 1.39±0.101 | 1.49±0.063 | 1.20±0.683 | 1.72±0.301 | 1.54±0.139 |
| Thymus | 0.54±0.145 | 0.55±0.078 | 0.59±0.140 | 0.50±0.168 | 0.39±0.097 | 0.41±0.123 |
| Testes | 3.25±0.133 | 3.41±0.180 | 3.36±0.059 | 3.39±0.221 | 3.51±0.142 | 3.39±0.189 |
| Epididymis | 1.14±0.081 | 1.16±0.056 | 1.21±0.118 | 1.14±0.092 | 1.32±0.065 | 1.35±0.104 |
| Prostate+ Seminal Vesicle | 3.14±0.289 | 2.64±0.103 | 2.85±0.321 | 2.84±0.375 | 3.78±0.420 | 3.61±0.457 |
| **Female (n=5)** | | | | | | |
| Liver | 10.33±0.757 | 9.73±0.852 | 9.89±1.115 | 9.49±1.527 | 10.08±0.879 | 10.17±0.540 |
| Kidneys | 1.90±0.220 | 2.12±0.213 | 2.01±0.171 | 1.92±0.262 | 2.01±0.139 | 1.95±0.075 |
| Adrenals | 0.08±0.011 | 0.08±0.014 | 0.08±0.017 | 0.08±0.002 | 0.08±0.009 | 0.08±0.013 |
| Spleen | 0.54±0.072 | 0.54±0.135 | 0.52±0.126 | 0.53±0.105 | 0.58±0.072 | 0.53±0.067 |
| Brain | 2.02±0.063 | 1.97±0.097 | 2.00±0.050 | 2.03±0.141 | 2.05±0.074 | 1.97±0.163 |
| Heart | 0.96±0.128 | 0.96±0.051 | 0.91±0.065 | 0.92±0.089 | 0.99±0.165 | 0.95±0.067 |
| Thymus | 0.47±0.037 | 0.40±0.132 | 0.49±0.194 | 0.41±0.131 | 0.40±0.064 | 0.44±0.058 |
| Ovaries | 0.15±0.022 | 0.15±0.020 | 0.13±0.018 | 0.15±0.019 | 0.13±0.027 | 0.16±0.014 |
| Uterus + Cervix | 0.69±0.235 | 0.91±0.136 | 0.80±0.240 | 0.89±0.203 | 0.94±0.343 | 0.92±0.246 |

*Note*: Data presented as Mean ± Standard Deviation (n = 5) and was statistically analysed by employing two-way ANOVA followed by Dunnett’s multiple comparison test. G1 (0); Vehicle control animals received 0.5% Methyl cellulose (10mL/kg/day), G2 (100), G3 (300), G4 (1000): Experimental animals received Renogrit at corresponding doses of 100, 300 and 1000 mg/kg/day for 28 consecutive days. G1R (0): Vehicle recovery group animals, observed 14 days after withdrawing 28 days 0.5% Methyl cellulose treatment. G4R (1000): Renogrit recovery group animals, observed 14 days after withdrawing 28 days Renogrit treatment.

Absolute organ weights from the experimental group animals revealed that Renogrit-administered rats, allocated to both the main and recovery groups, were comparable to those of their corresponding controls to demonstrate the systemic safety.

SUPPL. TABLE 9: Effect of Renogrit on relative organ weights represented as percentage of terminal brain weights.

| **Parameter** | **28 days Renogrit treatment (mg/kg/day)** | | | | **14 days recovery (mg/kg/day)** | |
| --- | --- | --- | --- | --- | --- | --- |
|  | **G1 (0)** | **G2 (100)** | **G3 (300)** | **G4 (1000)** | **G1R (0)** | **G4R (1000)** |
| **Males (n=5)** | | | | | | |
| Brain weight (g) | 2.16±0.0737 | 2.07±0.1071 | 2.05±0.0913 | 2.03±0.0623 | 2.11±0.1490 | 2.16±0.1011 |
| Liver | 753.62±89.63 | 737.28±97.03 | 909.48±141.64 | 843.49±136.95 | 963.09±175.08 | 831.07±68.91 |
| Kidneys | 147.66±9.16 | 144.75±9.86 | 166.56±14.05 | 164.77±14.78 | 179.40±20.28 | 168.17±21.77 |
| Adrenals | 3.28±0.33 | 2.69±0.93 | 2.97±0.53 | 3.56±0.78 | 3.93±0.78 | 3.25±0.62 |
| Spleen | 37.01±4.78 | 38.10±7.67 | 40.82±5.17 | 39.71±7.01 | 37.51±7.51 | 40.29±6.32 |
| Brain | 100.00±0.00 | 100.00±0.00 | 100.00±0.00 | 100.00±0.00 | 100.00±0.00 | 100.00±0.00 |
| Heart | 64.90±3.53 | 67.43±4.44 | 73.02±4.78 | 58.53±33.44 | 82.12±19.92 | 71.67±6.08 |
| Thymus | 24.92±6.54 | 26.74±4.73 | 29.11±7.57 | 24.72±8.63 | 18.48±4.92 | 19.28±5.80 |
| Testes | 150.45±7.64 | 165.01±7.04 | 164.38±7.78 | 166.81±14.33 | 166.67±15.02 | 158.40±16.12 |
| Epididymis | 52.80±3.39 | 56.06±1.59 | 59.00±3.79 | 56.34±5.86 | 62.56±5.54 | 63.14±6.37 |
| Prostate+ Seminal Vesicle | 145.04±8.92 | 127.78±6.80 | 139.12±12.09 | 139.90±20.31 | 179.44±23.33 | 167.94±19.08 |
| **Females (n=5)** | | | | | | |
| Brain weight (g) | 2.02±0.06 | 1.97±0.097 | 2.00±0.05 | 2.03±0.14 | 2.05±0.07 | 1.97±0.16 |
| Liver | 510.10±23.26 | 495.11±46.87 | 494.24±59.42 | 465.95±48.26 | 490.67±40.39 | 518.91±39.39 |
| Kidneys | 93.48±8.12 | 108.09±13.97 | 100.49±10.79 | 94.47±6.41 | 97.98±7.12 | 99.30±6.57 |
| Adrenals | 3.84±0.51 | 3.98±0.52 | 3.97±0.95 | 4.08±0.31 | 3.97±0.34 | 3.94±0.59 |
| Spleen | 26.51±3.44 | 27.75±8.13 | 26.00±6.91 | 26.09±3.49 | 28.37±3.16 | 26.94±2.66 |
| Brain | 100.00±0.00 | 100.00±0.00 | 100.00±0.00 | 100.00±0.00 | 100.00±0.00 | 100.00±0.00 |
| Heart | 47.12±5.23 | 48.91±3.17 | 45.72±4.17 | 45.14±2.54 | 48.09±8.29 | 48.43±4.40 |
| Thymus | 23.03±2.22 | 20.49±7.20 | 24.77±10.17 | 20.22±6.31 | 19.39±2.57 | 22.76±4.70 |
| Ovaries | 7.30±1.15 | 7.70±1.38 | 6.45±1.00 | 7.63±1.00 | 6.56±1.35 | 8.21±0.79 |
| Uterus + Cervix | 34.03±10.82 | 46.50±8.00 | 39.93±12.41 | 43.75±9.57 | 45.32±15.50 | 46.03±9.12 |

*Note*: Data presented as Mean ± Standard Deviation (n = 5) and was statistically analysed by employing two-way ANOVA followed by Dunnett’s multiple comparison test. G1 (0); Vehicle control animals received 0.5% Methyl cellulose (10mL/kg/day), G2 (100), G3 (300), G4 (1000): Experimental animals received Renogrit at corresponding doses of 100, 300 and 1000 mg/kg/day for 28 consecutive days. G1R (0): Vehicle recovery group animals, observed 14 days after withdrawing 28 days 0.5% Methyl cellulose treatment. G4R (1000): Renogrit recovery group animals, observed 14 days after withdrawing 28 days Renogrit treatment.

Relative organ weights w.r.t. the respective brain weight from the experimental group animals revealed that Renogrit-administered rats, allocated to both the main and recovery groups, were comparable to those of their corresponding controls to demonstrate the systemic safety.
